# The Level and Nature of Impairment on the Iowa Gambling Task Following Acquired Brain Injury: A Meta-analysis

**DOI:** 10.1007/s11065-025-09668-4

**Published:** 2025-06-14

**Authors:** Sammy Moore, Kristin Naragon-Gainey, Carmela F. Pestell, Rodrigo Becerra, Melissa T. Buelow, Danielle M. Fynn, Michael Weinborn

**Affiliations:** 1 School of Psychological Science, The University of Western Australia, 35 Stirling Highway, Crawley, Perth, WA 6009 Australia; 2 Department of Psychology, The Ohio State University, Columbus, USA

**Keywords:** Acquired brain injury, Traumatic brain injury, Executive functioning, Iowa Gambling Task, Decision-making

## Abstract

The Iowa Gambling Task (IGT) is a popular measure of risky decision-making, but to date, no formal quantitative reviews have been conducted, focused exclusively on IGT performance amongst individuals with acquired brain injury (ABI). Therefore, this meta-analytic study firstly explored performance differences between individuals with ABI vs controls. Second, we extended this comparison by investigating differences in IGT scoring and interpretive approaches (e.g., total score vs later block analysis). Finally, we explored potential IGT performance moderators (e.g., average age). A total of 25 studies, containing 39 samples (total *n* = 2188), were included. Overall findings suggested that the IGT is sensitive to the presence of ABI, particularly non-TBI and medically confirmed TBI, which becomes evident by block 2 of 5. Medium effect sizes were obtained for IGT total score, as well as indicators using later blocks only. Performance moderators such as population type and region influenced IGT performance, whilst average age, average education, and proportion of males did not. These results indicate that the IGT is sensitive to decision-making impairment following ABI, although we conclude that further research is needed to confirm the IGT’s ability to detect impairment relative to specific brain regions.

**Supplementary Information:**

The online version contains supplementary material available at 10.1007/s11065-025-09668-4.

## Introduction

Individuals diagnosed with an acquired brain injury (ABI) can experience significant physical, cognitive, and emotional dysfunction. ABI can occur as the result of traumatic brain injury (TBI; e.g., falls, motor accidents) or non-traumatic (e.g., stroke, encephalitis) injury (Cullen et al., 2008). ABI can impact the ability to work, study, engage in social activities, and live independently (Australian Institute of Health and Welfare (AIHW), 2007).

Executive functioning (EF) is an umbrella term used to describe a series of higher-order cognitive processes (Anderson et al., 2010) that are often impaired following ABI. Traditional EF measures have been argued to lack sensitivity to some types of neurobehavioral presentations associated with prefrontal cortex (PFC) injury. The Iowa Gambling Task (IGT) was subsequently developed to assess the neurobehavioral presentation of impaired decision-making (Bechara et al., 1994). The IGT has become a common measure of this component of EF across research and clinical settings. However, to date, no formal quantitative reviews specifically exploring the performance of individuals with brain injury on the IGT have been published. Therefore, this paper reviews the extant literature addressing the level and nature of performance on the IGT amongst individuals with ABI compared to controls.

### Hot Executive Functioning and Acquired Brain Injury

EFs describe various top-down processes essential for regulating behavior, mental and physical health, and social, cognitive, and psychological development (Diamond, 2013). Traditional definitions of EF include processes such as inhibition, planning, working memory, mental flexibility, and set shifting (Chan et al., 2008). However, EF is also conceptualised based on the degree to which these processes relate to emotion. Processes that occur in the absence of emotion (i.e., inhibition, working memory, set-shifting) are referred to as “cold” EF, whereas processes that include emotional aspects—such as social cognition, risky decision-making, and emotion regulation—are deemed “hot” EF (Chavez-Arana et al., 2018). These hot/cold processes have been associated with particular brain regions, primarily the PFC. For example, the ventromedial prefrontal cortex (VMPFC), the medial prefrontal cortex (MPFC), and the orbitofrontal cortex (OFC) have been associated with hot EF. In contrast, the dorsolateral prefrontal cortex (DLPFC), ventrolateral prefrontal cortex (VLPFC), and anterior cingulate cortex (ACC) have been associated with cold EF (Salehinejad et al., 2021). Subcortical regions thought to be involved in hot EF include the amygdala, insula, hippocampus, striatum, and brainstem (Salehinejad et al., 2021). However, implying that a single region is responsible for specific behaviors is overly simplistic, and instead, the neuroanatomical substrates of EF performance may be best thought of as an interrelated network (Nejati et al., 2018). This is fundamental to our understanding of hot and cold EFs, as many day-to-day tasks rely on input from multiple brain regions. For example, Wood and Worthington (2017) suggest that cold EF processes draw upon attention and memory to maintain social interactions. However, these interactions also rely on the ability to evaluate and interpret relevant behavioral and emotional cues—thus also activating neural substrates associated with hot EF (Bechara et al., 2000a, 2000b). Subsequently, deficits in one domain may have flow on effects to other domains. For example, reduced capacity to encode, monitor, or recall contents of working memory may result in hot EF processes taking over pre-planned goal-directed behaviors (cold EF processes)—resulting in impulsive dysregulated behaviors (Wood & Worthington, 2017).

Despite this, measures of cold EF are primarily used to assess dysfunction following an ABI, with hot EF largely neglected (Wood & Worthington, 2017). For instance, numerous studies have found that EF is a predictor of functional outcome post-ABI (Allanson et al., 2017; Spitz et al., 2012); however, these studies only focus on cold EF. This is problematic, as research has found that whilst patients with damage to the VMPFC might perform well on traditional *cold* EF measures, they may struggle with decision-making and other tasks that require emotional components in everyday life (Bechara, 2004). This suggests that solely relying on cold EF measures may inaccurately reflect everyday EF in people with ABI, potentially impacting diagnosis and treatment outcomes.

Whilst there is general consensus on the definition of cold EF within the literature (Miyake et al., 2000; Stuss & Alexander, 2007), processes such as emotion regulation, impulsivity, or decision-making are not always categorised as components of hot EF. For instance, when exploring the impact of dimensions such as impulsivity and emotional hyperactivation on functional outcomes, Rebetez et al. (2015) found that high levels of urgency lead to higher levels of emotional hyperactivation and thus poorer functional outcomes. However, they did not classify any of the domains measured as hot EF. This lack of consistency regarding what exactly constitutes hot EF may be a contributing factor to limited research and clarity in this area. Interestingly, this issue appears to be less prevalent in adolescent research, where hot EF has been a focal point of increased research interest (e.g., Kouklari et al., 2017; Poon, 2017). In addition, it should be noted that some conceptual disagreement remains, with some researchers arguing that hot EFs could be alternative labels for aspects of personality, reflecting a “jangle” fallacy, where the same construct is described using different terminology (Limpo & Olive, 2021).

A lack of research on hot EF following ABI in adults results in clinicians relying on cold EF measures to assess EF dysfunction (Wood & Worthington, 2017). However, recommendations regarding intervention may differ based on whether an individual has hot or cold EF deficits. Specifically, some have argued that in order to ensure a comprehensive neuropsychological profile, assessment of EF should routinely include hot EF measures in addition to cold EF (Chavez-Arana et al., 2018). To date, few published tasks specifically examine hot EF, and of those, few have normative data. One such task that has normative data and is commonly used in both clinical and research settings is the IGT.

### Iowa Gambling Task

The IGT was developed by Bechara et al. (1994) to identify dysfunction in individuals with VMPFC damage who often perform well on traditional cognitive tasks but display impairments for decision-making and other skills with emotional components (Eslinger & Damasio, 1985). Accumulating research on the IGT has supported its sensitivity to decision-making impairment amongst those with ABI (including those with VMPFC lesions, but also those with injury in other regions) (Buelow & Suhr, 2009).

Whilst the original IGT was conducted in person, the clinical version (available since 2007) is conducted via computer and asks participants to select one card at a time from four decks (A, B, C or D), over 100 trials. Participants are told minimal information, e.g., “that they will be given $2000 (play money) to begin” with and “must aim to lose as little money as possible.” After a card is selected, feedback about how much money they have won and lost is provided. All decks result in an immediate “win,” but some also result in a “loss” of differing amounts. These gain–loss values are different for each deck, with some losses outnumbering wins. Participants are told that some decks are better than others but must infer which ones these are throughout the task. To perform well, participants must abandon large short-term wins and instead implement a long-term strategy. This requires the selection of decks C and D—as these are the “good” decks that lead to greater monetary gains with smaller losses over time. A total net score is calculated by combining the frequency of good deck selections (C + D) and deducting the frequency of bad deck selections (A + B) for all 100 trials (across 5 blocks of 20 trials each). At the start of the IGT, participants typically trial all four decks within the first or second blocks and move towards selecting from more advantageous decks later in the task (Bechara et al., 2000a, 2000b). Dunn et al. (2006) refer to this change in strategy as “acquiring” the IGT, with controls acquiring the IGT much faster than individuals with decision-making impairments.

Since its inception, there have been numerous approaches to scoring, presenting, and interpreting the findings from the IGT. Some researchers use total net scores (Balagueró et al., 2016; Homaifar et al., 2012); others use net block scores (Fonseca et al., 2012); some provide variations based on loss frequency, such as B + D or A + C (e.g., Besnard et al., 2015), or frequency of deck selections (Fellows & Farah, 2005; MacPherson et al., 2009; Steingroever et al., 2013). The most common approach is to provide the net raw total and the five block scores ([C + D] − [A + B]). The inclusion of block scores is important, as research, first by Brand et al. (2007), highlighted that the IGT might be measuring two aspects of decision-making—with earlier blocks measuring “ambiguity” as participants begin to figure out the task, and later blocks measuring “risk” as they infer the cost–benefit of each deck. As such, the inclusion of both block and total scores provides insight into performance *across* the IGT, consistent with recommendations by Buelow and Suhr (2009) and Levine et al. (2005).

Whilst the IGT has sometimes been conceptualised as tapping into cold EF (e.g., set-shifting and decision-making; Gansler 2011), it is fundamentally considered to be a hot EF task that primarily measures risky decision-making (Bull et al., 2015; Salehinejad et al., 2021). A thorough review paper by Buelow and Suhr (2009) provides evidence for the construct validity of the IGT in ABI. With regard to concurrent and divergent validity, the IGT has demonstrated relatively low correlations with other cold EF tasks (Toplak et al., 2010), although a possible explanation is that the task was created to measure factors that were missed by traditional EF tasks (Bechara 1994). However, factor analyses by Buelow and Blaine (2015) found that the IGT does not appear to load with other hot EF tasks, such as the Balloon Analogue Risk Task (BART) or the Columbia Card Task (CCT-Hot), suggesting that the IGT measures a construct of decision-making different to those tasks as well.

Regarding the reliability of the IGT, numerous studies have found that test–retest values are low to moderate for controls—as once the task is acquired, it is no longer novel (Buelow & Barnhart, 2018; Cardoso et al., 2010). However, in clinical samples, no practice effects or recovery have been observed on repeated administrations of the task (Waters-Wood et al., 2012).

### Iowa Gambling Task Performance Amongst Individuals with Acquired Brain Injury

Whilst several meta-analytic papers have been conducted on the level and nature of performance on the IGT within clinical populations, none has been specific to ABI (Kovács et al., 2017; Rotge et al., 2017). Dunn et al. (2006) provided a qualitative summary of the usage of the IGT in a variety of clinical populations including ABI and concluded that the IGT is sensitive to the presence of brain injury. Specifically, impairment was associated with brain injuries affecting the VMPFC, insula, amygdala, and somatosensory cortex. However, they primarily reviewed papers from Bechara and associates from 1994 to 2004, which included small and at times overlapping samples (Hochman et al., 2010).

Since then, other research teams have found evidence supporting the IGT’s sensitivity to ABI more globally. Both MacPherson et al. (2009) and Fellows and Farah (2005) explored IGT performance in individuals with prefrontal brain damage, comparing individuals with primarily VMPFC lesions to either non-VMPFC lesions (MacPherson et al., 2009) or to DLPFC lesions (Fellows & Farah, 2005). Compared to controls, both studies found ABI groups performed worse on the IGT regardless of lesion location, with no significant differences between the ABI groups.

Similar findings have also been reported in ABI samples with damage outside of prefrontal regions. Cardoso et al. (2014) examined stroke patients categorised by damage to either frontal or cerebellar regions. Compared to controls, they found that both groups displayed impaired performance on the IGT, measured by total net score. Again, no significant difference in the total net scores was observed between the clinical groups. Contrary to these findings, Besnard et al. (2017) found no difference in IGT performance between individuals with prefrontal lesions (*n* = 15) and controls (*n* = 30). This was the case for both net total and block scores (although the prefrontal group performed better on block 2). Another study, by Wang et al. (2021), examined performance of VMPFC and DLPFC samples versus controls and found that only the VMPFC sample performed significantly worse as measured by IGT total score. Additionally, individuals with ABI also appear to differ in their patterns of performance whilst completing the IGT. Examples from aforementioned studies include MacPherson et al. (2009) who found that ABI patients performed more poorly in later blocks compared to controls. Interestingly, Cardoso et al. (2014) found that whilst both the frontal and cerebellar patients performed worse than controls in later blocks, the cerebellar patients made slightly more advantageous deck selections compared to the frontal group. These findings suggest that the learning curves differ between the two clinical groups, and therefore, there may be different underlying mechanisms that account for impaired performance on the task. Thus, the extant literature appears to preliminarily support the sensitivity of the IGT to the presence of ABI, regardless of lesion location. Furthermore, there appears to be value in analysing block, as well as total scores. However, prior studies are limited by small sample sizes, ranging from 6 to 29 participants. In addition, the literature is constrained by reporting different outcome measures (i.e., advantageous deck selections vs net block scores) which significantly hinders comparison across studies. In terms of moderating factors that influence IGT performance, some evidence suggests that males typically perform better than females (Bolla et al., 2004; Weller et al., 2010). However, these findings have not been found in ABI samples (MacPherson et al., 2009; Scheffer et al., 2011).

### Aims and Hypotheses

Given the above findings, the present meta-analysis aims to answer the following questions: Firstly, what are the common patterns of IGT performance for an adult with an ABI (regardless of lesion location or severity), and does it significantly differ from controls? Previous findings suggest that the ABI groups will perform more poorly than non-clinical groups.

Secondly, are block scores and total scores similarly impaired amongst individuals with ABI on the IGT? Specifically, do composite scores derived from later blocks provide a larger effect size compared to the total score? Combinations of blocks 2, 3, 4, and 5 *and* 3, 4, and 5 will be calculated and compared between groups with the expectation that they will indeed provide a greater effect size and therefore reflect higher levels of hot EF dysfunction than the total score alone. This is consistent with the notion that controls acquire the IGT faster (Dunn et al., 2006).

If feasible, moderator analyses will be conducted to explore factors that may be influencing IGT performance. These include injury characteristics (such as lesion location and injury severity) and demographic characteristics (such as proportion of males, average age, and average education).

## Method

This meta-analysis was conducted in accordance with the Preferred Reporting Items for Systematic Reviews and Meta-Analyses guidelines (PRISMA; Moher et al., 2009; see Tables S6 and S7) and was preregistered (CRD42021279100) on 27 September 2021 with the international register of systematic reviews (PROSPERO, Booth et al., 2011).^1^

### Literature Search

An extensive search of the literature was undertaken on 28 September 2021, sourcing studies that utilised the IGT in ABI samples. An updated search was conducted on 22 March 2023. The following online databases were searched: EMBASE, EBSCO MEDLINE, Proquest, PsycINFO, Scopus, and Web of Science with the following search terms: *(brain injury OR acquired brain injury OR head injury OR brain ischemia OR traumatic brain injury OR stroke OR TBI OR ABI) AND (hot executive function OR Iowa gambling task OR hot cognition OR gambling task OR risky decision-making OR somatic marker hypothesis)*. Broad selection criteria were used, and no date restrictions were applied to obtain as many sources as possible. In conjunction with the electronic search, reference lists from papers that met eligibility criteria were hand searched to broaden the scope. Grey literature sources (e.g., dissertations, conference presentations) were also considered if identified via the above methods.

### Study Selection

Inclusion criteria were studies using the IGT in ABI samples, written in English or Spanish (author RB is bilingual), and using adult samples (16–75 years old). When studies appeared to meet the criteria but did not report the necessary statistics, the authors were contacted to request the data. Both the original version of the IGT (Bechara et al., 1994) and the computerised version (Bechara, 2007) were accepted, as no differences have been found between the tasks (Bechara et al., 2000a, 2000b). Modifications of the IGT were accepted, but only for minor alterations such as language and currency adaptions. Additionally, variations in the proportion of winnings were allowed—such as when the total pot started with $0 (Schneider & Parente, 2006) instead of $2000 as per the original. To account for statistical heterogeneity and subsequent subgroup analysis, we coded for the presence of modified versions of the task. Confirmed brain injury (via medical records and/or clinical assessment) and self-reported head injury were accepted and coded for moderator analyses.

Articles were excluded if the study did not have human participants or if participants were selected based upon addiction, neurodegenerative, psychiatric, or other non-ABI conditions. Finally, theoretical, commentary, or discussion papers were omitted.

### Quality Control/Risk of Bias

To ensure methodological quality, author DF replicated portions of the search process and conducted the risk of bias analysis. For the literature search, author SM randomly generated 20% of the papers at both the abstract and full-text phases of the PRISMA process for DF to review independently. The results generated from DF and SM were compared and discussed for consensus with no disagreements occurring (*κ* = 1). In terms of the data extraction process, SM presented the data that was feasible to be extracted to the supervisory team (MW, CP, KNG, and RB), which was reviewed and accepted with no disagreements.

Risk of bias was calculated using the Quality Assessment of Diagnostic Accuracy Studies 2 criteria (QUADAS-2) (Whiting et al., 2011). Four domains that assessed patient selection, index test, reference standard, and flow and timing were utilised. Signalling questions and domain scores for each study are found in Table S1. Author DF independently conducted risk of bias assessments on 20% of randomly selected studies. There was initial moderate agreement between the two raters (*κ* = 0.0.581), and disagreements were resolved after discussion.

### Data Extraction

Means and standard deviations (SD) were extracted from the following variables: demographic information (i.e., average age, average education, proportion of males) and IGT scores (i.e., either raw net block scores and/or total scores [C + D-A + B]). It was deemed that total net raw score ([C + D]-[A + B]) would be the best index to use for the IGT, as it is the most utilised metric within the literature. Where possible, the net raw scores across the five blocks were also obtained and extracted. Additional information such as study location (i.e., country), population type (i.e., clinical classification), IGT version (i.e., original vs modified), and TBI history source (i.e., TBI-self-report vs TBI-confirmed) was collected for subsequent analysis. Whilst injury information such as time since injury, injury severity, and lesion location were desired, insufficient data were available. Papers that included more than one sample in their study were treated as separate samples for analysis. Data extraction was managed using Microsoft Excel.

### Data Cleaning

Data presented in Spanish and Portuguese were translated using a free online service (DeepL Translator, n.d) and was double-checked by author RB. Where data appeared to be incorrect, steps were taken to amend it where reasonable. For example, the values reported by Xi et al. (2011) were assumed to be SD not the standard error of the mean (SEM) and were treated as such for analysis.^2^ Additionally, for Scheffer et al. (2011), the SDs for blocks 4 and 5 were amended to positive values given that negative SD are out of bounds.^3^ The combination of block means and SDs was calculated using the guide from Higgins et al. (2022).

Missing data were removed case-wise where relevant (i.e., block vs total analyses). For the six papers that did not have control data, sensitivity analysis revealed that the most appropriate control data to use was from studies with the same country of origin (Deeks et al., 2022). In the case of Sigurdardottir et al. (2010), the dataset from Fonseca et al. (2012) was selected as it was closer to population, average age, average education, and proportion of males, and sensitivity analysis was conducted to confirm this. To prevent the control sample size from Barnhart and Buelow (2021) from upwardly biasing the meta-analysis, it was winsorised from 1547 to 66, similar to the method utilised by Fynn et al. (2021).^4^

Some data had to be further adapted for moderation analyses. For example, the number of countries was too large and was subsequently classified by regions (Asia Pacific, Europe, Latin America, and North America) based on criteria from Pew Research Centre (2013). The participant clinical population (presented in Table 1) was too broad, thus categorised into four groups: (1) *non-TBI*, (2) *TBI-confirmed*, (3) *TBI-self-report*,^5^ and (4) *other* (i.e., mixed etiology or unclear etiology), which included a heterogenous sample of *n* = 3 and was therefore excluded from the population type subgroup analysis. Finally, to handle missing data on moderator variables, we employed multiple imputation using the mice package in R (van Buuren & Groothuis-Oudshoorn, 2011). Specifically, we utilised predictive mean matching for imputing missing continuous variables. We imputed 14 missing values across age, gender, and education. Despite the relatively small number of studies, multiple imputation is appropriate for handling missing predictor data in meta-regression models (Pigott, 2001), offering more accurate and unbiased estimates than methods like listwise deletion, which can reduce statistical power and introduce bias (Pigott, 2001). This approach enabled us to include all studies in a multivariable meta-regression model, addressing concerns about missing values impacting the results. Sensitivity analyses indicated that the imputation did not unduly influence our conclusions.^6^

**Table 1:** Study information and demographics from selected papers in the meta-analysis

Participant population	Group	Population type	IGT version	Region	Clinical				Controls			
					*n*	% male	Average age (SD)	Average education (SD)	*n*	% male	Average age (SD)	Average education (SD)
Allanson (2019)												
Non-TBI	Clinical vs controls	Non-TBI	Original	Asia Pacific	33	39.00	46.64 (13.51)	12.97 (2.39)	67	59.70	40.51 (17.47)	13.66 (2.35)
TBI (all types)	Clinical vs controls	TBI ‒ Confirmed	Original	Asia Pacific	33	78.00	39.18 (15.52)	12.83 (2.98)	67	59.70	40.51 (17.47)	13.66 (2.35)
Barnhart and Buelow (2021)												
TBI (all types)	Clinical vs controls	TBI ‒ Self-Report	Original	North America	52	53.85	19.00 (3.02)	N/A	66	41.70	18.88 (2.58)	N/A
Besnard et al. (2017)												
Prefrontal lesions	Clinical vs controls	Other	Original	Europe	15	86.67	53.80 (18.90)	9.60 (1.80)	30	26.67	55.10 (22.60)	10.40 (2.70)
Brenner et al. (2015)												
Moderate to severe TBI (without suicide attempt)	Clinical vs controls	TBI ‒ Confirmed	Original	North America	50	100.00	53.00 (8.50)	N/A	48	83.33	54.20 (7.60)	N/A
Moderate to severe TBI (with suicide attempt)	Clinical vs controls	TBI ‒ Confirmed	Original	North America	22	86.36	49.50 (10.30)	N/A	12	66.67	51.70 (12.00)	N/A
Canto Pech et al. (2007)												
Severe TBI	Clinical	TBI ‒ Confirmed	Modified	Europe	13	75.00	26.54 (6.76)	11.25 (2.59)	31	54.84	50.00 (11.10)	11.10 (3.20)
Cardoso et al. (2014)												
Cerebellar stroke	Clinical vs controls	Non-TBI	Modified	Latin America	9	44.44	64.10 (6.27)	9.67 (4.18)	18	23.00	59.28 (10.25)	12.08 (6.18)
Frontal stroke	Clinical vs controls	Non-TBI	Modified	Latin America	9	44.44	57.70 (8.62)	11.30 (2.87)	18	23.00	59.28 (10.25)	12.08 (6.18)
Cardoso et al. (2015)												
Stroke	Clinical	Non-TBI	Modified	Latin America	99	N/A	N/A	N/A	18	23.00	59.28 (10.25)	12.08 (6.18)
Clark et al. (2003)												
Focal frontal lesions—left hemisphere	Clinical vs controls	Non-TBI	Original	Europe	22	59.09	53.30 (11.00)	N/A	21	47.62	50.70 (5.90)	N/A
Focal frontal lesions—right hemisphere	Clinical vs controls	Non-TBI	Original	Europe	24	33.33	54.30 (11.00)	N/A	21	47.62	50.70 (5.90)	N/A
Escartin et al. (2012)												
Sub-arachnoid hemorrhage (treatment 1)	Clinical vs controls	Non-TBI	Modified	Europe	20	65.00	46.20 (10.20)	10.50 (3.70)	31	54.84	50.00 (11.10)	11.10 (3.20)
Sub-arachnoid hemorrhage (treatment 2)	Clinical vs controls	Non-TBI	Modified	Europe	20	50.00	53.00 (8.80)	9.65 (2.70)	31	54.84	50.00 (11.10)	11.10 (3.20)
Fonseca et al. (2012)												
TBI (all types)	Clinical vs controls	TBI-confirmed	Modified	Latin America	16	75.00	37.31 (13.65)	10.50 (3.48)	16	56.25	32.88 (13.09)	12.44 (4.20)
Hochman et al. (2010)												
Prefrontal lesions	Clinical	Other	Original	North America	11	81.82	50.38 (16.14)	11.46 (3.18)	48	83.33	54.20 (7.60)	N/A
Homaifar et al. (2012)												
TBI (all types with suicide attempt)	Clinical	TBI-confirmed	Original	North America	18	83.00	49.60 (8.40)	N/A	48	83.33	54.20 (7.60)	N/A
TBI (all types without suicide attempt)	Clinical	TBI-confirmed	Original	North America	29	100.00	52.20 (10.60)	N/A	48	83.33	54.20 (7.60)	N/A
Krpan et al., (2011a, 2011b)												
Mild TBI	Clinical vs controls	TBI-self-report	Original	North America	10	30.00	54.10 (13.32)	16.00 (4.06)	24	42.00	38.75 (17.48)	15.79 (3.38)
Moderate to severe TBI	Clinical vs controls	TBI-self-report	Original	North America	17	64.70	39.35 (13.87)	15.18 (2.90)	24	42.00	38.75 (17.48)	15.79 (3.38)
Levine et al. (2005)												
TBI (all types)	Clinical vs controls	TBI-confirmed	Original	North America	71	59.20	30.82 (10.40)	14.21 (2.53)	19	21.05	29.60 (8.90)	15.10 (2.00)
Minjoz et al. (2023)												
ABI	Clinical	Non-TBI	Original	Europe	21	47.62	58.24 (10.22)	N/A	30	26.67	55.10 (22.60)	N/A
TBI (all types)	Clinical	TBI-confirmed	Original	Europe	5	80.00	34.20 (8.58)	N/A	30	26.67	55.10 (22.60)	N/A
Ouerchefani et al. (2017)												
Focal frontal lesions	Clinical vs controls	Other	Modified	Europe	27	85.19	41.33 (12.96)	7.30 (2.11)	34	88.24	39.03 (12.86)	8.15 (2.70)
Robb and Good (2011)												
Mild TBI	Clinical vs controls	TBI-self-report	Original	North America	10	40.00	22.90 (4.98)	13.80 (1.48)	23	60.00	21.65 (3.86)	13.96 (1.36)
Robb and Good (2014)												
Mild TBI	Clinical vs controls	TBI-self-report	Original	North America	32	46.88	20.78 (2.38)	13.91 (1.53)	43	48.84	20.77 (2.98)	13.86 (1.39)
Moderate TBI	Clinical vs controls	TBI-self-report	Original	North America	9	77.78	22.44 (5.46)	14.33 (2.00)	43	48.84	20.77 (2.98)	13.86 (1.39)
Scheffer et al. (2011)												
Stroke—female	Clinical	Non-TBI	Modified	Latin America	9	0.00	60.44 (11.57)	11.27 (5.86)	18	23.00	59.28 (10.25)	12.08 (6.18)
Stroke—male	Clinical	Non-TBI	Modified	Latin America	10	100.00	60.90 (8.93)	9.85 (4.46)	18	23.00	59.28 (10.25)	12.08 (6.18)
Sigurdardottir et al. (2010)												
Mild TBI	Clinical	TBI-confirmed	Original	Europe	40	63.00	35.90 (11.40)	14.30 (2.50)	16	56.25	32.88 (13.09)	12.44 (4.20)
Moderate TBI	Clinical	TBI-confirmed	Original	Europe	34	74.00	33.50 (10.80)	12.50 (3.00)	16	56.25	32.88 (13.09)	12.44 (4.20)
Severe TBI	Clinical	TBI-confirmed	Original	Europe	41	76.00	28.50 (10.40)	12.60 (1.90)	16	56.25	32.88 (13.09)	12.44 (4.20)
Van Noordt et al. (2010)												
Mild TBI	Clinical vs controls	TBI-self-report	Original	North America	18	N/A	20.39 (3.03)	12.94 (1.21)	26	N/A	19.35 (1.57)	12.96 (1.31)
Wang et al. (2021)												
DLPFC	Clinical vs controls	Non-TBI	Modified	Asia Pacific	29	N/A	48.24 (6.87)	10.45 (2.03)	32	N/A	21.34 (6.97)	10.34 (1.84)
VMPFC	Clinical vs controls	Non-TBI	Modified	Asia Pacific	27	N/A	49.59 (6.26)	10.19 (1.67)	32	N/A	21.34 (6.97)	10.34 (1.84)
Xi et al. (2011)												
DLPFC	Clinical vs controls	TBI-confirmed	Modified	Asia Pacific	14	42.86	32.43 (8.87)	7.78 (2.97)	30	46.67	34.76 (9.57)	9.50 (2.92)
VMPFC	Clinical vs controls	TBI-confirmed	Modified	Asia Pacific	16	62.50	37.06 (10.19)	8.94 (3.28)	30	46.67	34.76 (9.57)	9.50 (2.92)
Zhang (2017)												
Mild TBI	Clinical vs controls	TBI-confirmed	Original	North America	47	61.70	41.32 (11.47)	16.98 (2.47)	30	60.00	41.83 (13.49)	16.40 (2.31)
Zinchenko and Enikolopova (2017)												
Right frontal tumors	Clinical vs controls	Non-TBI	Original	Europe	12	75.00	34.00 (12.37)	N/A	21	57.00	29.86 (11.70)	N/A

Note. *IGT* Iowa Gambling Task, *ABI* acquired brain injury, *TBI* traumatic brain injury, *DLPFC* dorsolateral prefrontal cortex, *VMPFC* ventromedial prefrontal cortex

### Data Analysis

Given the presence of multiple samples from the same authors in our dataset, we performed multivariate meta-analyses (Cheung, 2019; Viechtbauer, 2010) to account for shared variance. All subsequent analyses were conducted using a multivariate approach.^7^ Analyses were conducted using the “metafor” package in R (R Core Team, 2024). As considerable between-study heterogeneity was expected, a random-effects model was used to combine effect sizes. This also allows the results to be generalised to studies that are not included in the meta-analysis. Effect sizes were calculated using the standardised mean difference between the clinical and control groups (Hedges’ *g*; Hedges, 1981).^8^ A variance–covariance matrix was constructed to model the dependency between effect sizes from the same study. The models were then fitted using the restricted maximum likelihood estimator (REML; Jackson et al., 2011) to capture the hierarchical structure of the data. Heterogeneity was assessed using two methods: Cochran’s *Q*-test, which tests for the presence of heterogeneity amongst studies by calculating the weighted sum of squares (Cochran, 1954); and the ${I}^{2}$ statistic, which quantifies the percentage of variability across studies that is due to heterogeneity rather than sampling error. Lower scores of ${I}^{2}$ represent low levels of heterogeneity (Higgins & Thompson, 2002). A significance level of < 0.05 was used for all analyses (i.e., standard meta-analysis, combined, outlier, publication bias, and heterogeneity statistics).

To examine the first hypothesis regarding ABI performance on the IGT, a total of six meta-analytic comparisons were conducted using IGT total and scores for each of the five IGT blocks. To investigate the influence of IGT scoring approaches (hypothesis two), additional meta-analyses were conducted for the combinations of blocks 2–5 and blocks 3–5. To assess potential differences between obtained pooled effect sizes for each IGT indicator (total score vs combination scores), the effect size for each indicator was assessed against the 95% CI range of the comparison indicator. To explore the influence of performance moderators on IGT scores, subgroup analysis was conducted for all categorical variables, and meta-regression was undertaken for continuous variables. For simplification, moderator analyses were conducted using total IGT scores only. The subgroup analyses explored population type (i.e., *non-TBI*, *TBI-confirmed*, *TBI-self-report*), IGT version (i.e., *modified*, *original*), and geographical region (i.e., *Asia Pacific*, *Europe*, *Latin America*, and *North America*), whilst meta-regression was undertaken to explore average *age*, *sex* (i.e., percent male), and average years of *education*.

To explore the sensitivity of meta-analytic results to extreme values, outlier analyses were performed for each of the five blocks and total scores. Residuals were calculated using the rstandard() function from the metafor package in R and then filtered based on whether they significantly deviate from the meta-analysis model predictions (Harrer et al., 2021). Specifically, studies with residuals exceeding ± 1.96 were flagged as potential outliers. These cases were then removed, and the meta-analysis was rerun without them to evaluate their impact on the overall results. Lastly, following recommendations from Harrer et al. (2021), publication bias was assessed via visual inspection of the funnel plot (Sterne et al., 2011) and the Precision-Effect Test and Precision-Effect Estimate with Standard Errors (PET-PEESE) method. PET-PEESE corrects for small-study effects by adjusting the relationship between standard errors and effect sizes (Stanley & Doucouliagos, 2014). In this method, the PET component tests for the presence of a true effect by regressing effect sizes on their standard errors. If the PET indicates a significant effect (i.e., rejects the null hypothesis of no effect), the PEESE component is then applied to provide a bias-corrected estimate by regressing effect sizes on the squared standard errors. Thus, PET-PEESE serves as a conditional estimator, with the choice between PET and PEESE estimates depending on the PET results (Stanley & Doucouliagos, 2014).^9^

## Results

### Study Inclusion and Characteristics

**Fig. 1:**
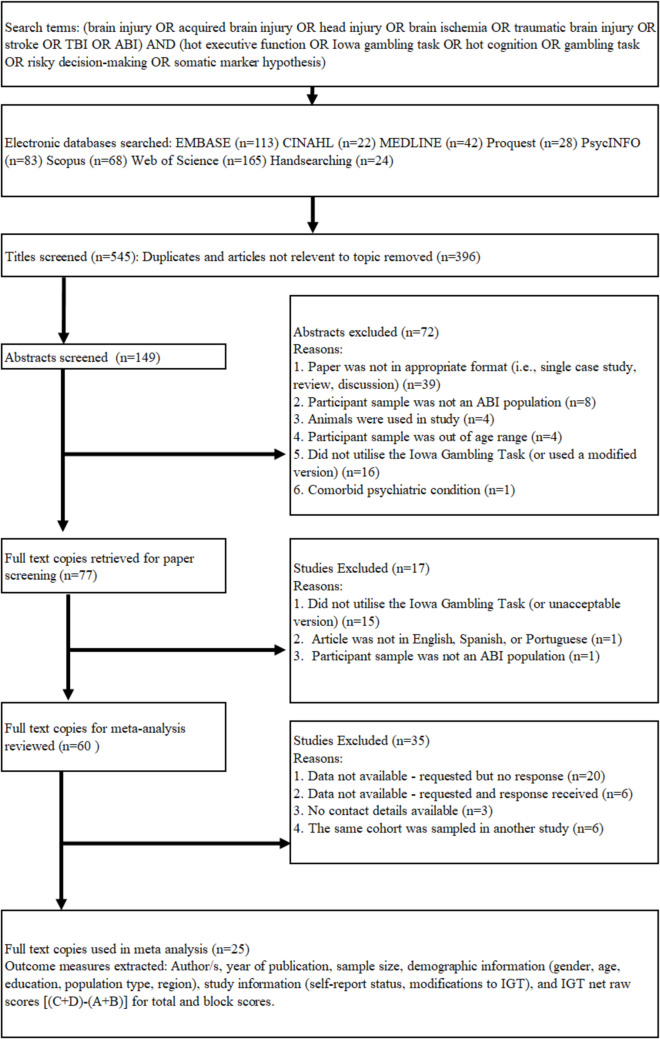
Preferred Reporting Items for Systematic Reviews and Meta-Analyses (PRISMA) study selection process

Following the removal of duplicates, a total of 149 papers were screened. Of those, 60 met the inclusion criteria. Forty-nine papers did not report all necessary data for analyses, and their authors were contacted for additional information. Of those, eight authors provided necessary statistics for analyses (Allanson, 2019; Barnhart & Buelow, 2021; Besnard et al., 2017; Hochman et al., 2010; Krpan et al., 2011a; Robb & Good, 2011, 2014; Van Noordt et al., 2010).^10^ Six papers were excluded as their samples were used in other included studies (Al-Khindi et al., 2010; Bechara et al., 1994, 1996, 2000a, 2000b; Besnard et al., 2015; Robb & Good, 2012). Additionally, 15 papers were excluded as IGT results were not presented in the appropriate format. For example, Bonatti et al. (2008) and Fellows and Farah (2005) used frequency of advantageous choices (C + D) whilst Manes et al. (2002) used frequency of risky choices (A + B). Attempts were made to extract the data from graphs (when there was no response from author contact), but this was unsuccessful. At the final stage, a total of 25 studies were included, with a total of 39 samples for analysis, including sub-sets within the same paper (Table 2). These comprised of 20 published journal articles, 2 theses, and 3 conference proceedings. See Fig. 1 for PRISMA study selection process.

**Table 2:** Iowa Gambling Task data from studies used in the meta-analysis

Participant population	Clinical							Controls				
	Block 1 (SD)	Block 2 (SD)	Block 3 (SD)	Block 4 (SD)	Block 5 (SD)	Total (SD)	Block 1 (SD)	Block 2 (SD)	Block 3 (SD)	Block 4 (SD)	Block 5 (SD)	Total (SD)
Allanson (2019)												
Non-TBI	− 3.09 (5.77)	3.15 (8.32)	3.94 (9.35)	4.55 (9.67)	3.09 (10.32)	12.21 (29.32)	− 1.07 (8.81)	7.01 (8.30)	9.58 (8.21)	7.91 (10.84)	8.42 (10.51)	31.85 (28.07)
TBI (all types)	− 3.39 (6.43)	1.27 (6.74)	0.30 (8.32)	0.97 (8.41)	1.82 (10.61)	0.42 (24.11)	− 1.07 (8.81)	7.01 (8.30)	9.58 (8.21)	7.91 (10.84)	8.42 (10.51)	31.85 (28.07)
Barnhart and Buelow (2021)												
TBI (all types)	− 3.02 (6.40)	0.58 (6.74)	1.52 (8.40)	1.33 (6.93)	1.63 (9.26)	2.04 (23.48)	− 2.82 (5.76)	0.19 (6.54)	0.87 (7.85)	1.38 (8.79)	1.54 (9.08)	1.12 (26.25)
Besnard et al. (2017)												
Prefrontal lesions	− 1.33 (8.30)	9.73 (8.68)	7.87 (7.50)	8.80 (9.44)	11.47 (6.99)	36.53 (23.11)	− 2.80 (3.35)	4.73 (7.62)	6.93 (7.14)	8.63 (8.34)	8.03 (9.25)	25.53 (22.14)
Brenner et al. (2015)												
Moderate to severe TBI (without suicide attempt)	− 1.70 (7.20)	0.00 (8.50)	0.36 (9.70)	1.50 (8.90)	1.50 (8.60)	1.70 (26.60)	− 2.60 (6.00)	0.79 (7.20)	0.88 (8.00)	3.50 (8.90)	2.80 (8.40)	5.30 (25.50)
Moderate to severe TBI (with suicide attempt)	− 1.00 (6.60)	0.09 (6.60)	− 1.20 (6.40)	− 0.09 (9.30)	− 2.00 (8.00)	− 4.20 (27.70)	− 2.20 (7.50)	− 1.70 (7.20)	− 0.83 (7.30)	3.80 (8.10)	4.30 (10.30)	3.50 (24.50)
Canto Pech et al. (2007)												
Severe TBI	− 3.00 (3.86)	− 1.00 (4.22)	− 0.83 (7.70)	0.00 (9.91)	− 3.33 (6.62)	− 8.17 (22.08)	− 3.94 (6.01)	1.97 (8.14)	3.29 (8.95)	4.10 (9.79)	6.90 (10.03)	11.10 (30.56)
Cardoso et al. (2014)												
Cerebellar stroke	− 0.22 (1.85)	− 1.78 (2.10)	1.33 (5.47)	5.56 (6.54)	0.67 (7.34)	3.78 (13.17)	− 0.44 (5.20)	3.44 (6.31)	7.44 (7.41)	6.00 (9.10)	5.89 (9.03)	23.00 (19.23)
Frontal stroke	− 1.56 (5.45)	0.67 (4.24)	− 4.44 (6.14)	− 3.33 (6.63)	− 2.89 (8.19)	− 16.44 (21.48)	− 0.44 (5.20)	3.44 (6.31)	7.44 (7.41)	6.00 (9.10)	5.89 (9.03)	23.00 (19.23)
Cardoso et al. (2015)												
Stroke	− 1.66 (4.00)	− 0.93 (4.34)	− 0.28 (5.26)	− 0.07 (7.24)	− 1.19 (7.56)	− 3.97 (19.12)	− 0.44 (5.20)	3.44 (6.31)	7.44 (7.41)	6.00 (9.10)	5.89 (9.03)	23.00 (19.23)
Clark et al. (2003)												
Focal frontal lesions—left hemisphere	N/A	N/A	N/A	N/A	N/A	7.00 (23.20)	N/A	N/A	N/A	N/A	N/A	23.60 (25.00)
Focal frontal lesions—right hemisphere	N/A	N/A	N/A	N/A	N/A	−9.14 (18.10)	N/A	N/A	N/A	N/A	N/A	23.60 (25.00)
Escartin et al. (2012)												
Sub-arachnoid hemorrhage (treatment 1)	− 1.40 (5.03)	− 1.10 (5.21)	0.00 (7.81)	2.90 (10.29)	− 0.90 (10.18)	− 0.50 (22.30)	− 3.94 (6.01)	1.97 (8.14)	3.29 (8.95)	4.10 (9.79)	6.90 (10.03)	11.10 (30.56)
Sub-arachnoid hemorrhage (treatment 2)	− 3.60 (5.82)	− 2.80 (6.37)	− 1.90 (7.90)	− 2.40 (11.41)	0.10 (7.77)	− 10.60 (25.95)	− 3.94 (6.01)	1.97 (8.14)	3.29 (8.95)	4.10 (9.79)	6.90 (10.03)	11.10 (30.56)
Fonseca et al. (2012)												
TBI (all types)	− 3.63 (5.62)	0.38 (6.24)	− 0.50 (3.05)	0.00 (3.09)	− 1.13 (3.79)	− 4.88 (11.95)	− 3.13 (6.81)	0.50 (6.42)	1.13 (9.68)	− 2.88 (11.26)	0.88 (8.45)	− 3.50 (25.67)
Hochman et al. (2010)												
Prefrontal lesions	− 6.67 (6.11)	− 1.17 (5.01)	1.67 (8.04)	− 5.50 (8.74)	− 3.50 (11.51)	− 15.17 (22.39)	− 2.60 (6.00)	0.79 (7.20)	0.88 (8.00)	3.50 (8.90)	2.80 (8.40)	5.30 (25.50)
Homaifar et al. (2012)												
TBI (all types with suicide attempt)	N/A	N/A	N/A	N/A	N/A	12.30 (33.80)	N/A	N/A	N/A	N/A	N/A	5.30 (25.50)
TBI (all types without suicide attempt)	N/A	N/A	N/A	N/A	N/A	17.30 (29.30)	N/A	N/A	N/A	N/A	N/A	5.30 (25.50)
Krpan et al., (2011a, 2011b)												
Mild TBI	− 2.60 (4.22)	0.00 (4.11)	0.40 (5.15)	− 3.20 (4.73)	− 1.60 (4.97)	− 7.00 (9.58)	− 4.17 (3.88)	− 1.83 (5.65)	0.70 (6.81)	1.04 (11.19)	2.35 (11.69)	− 1.83 (29.45)
Moderate to severe TBI	− 1.65 (4.37)	− 1.18 (4.13)	− 1.65 (7.94)	− 1.88 (6.76)	− 0.35 (8.28)	− 6.82 (27.04)	− 4.17 (3.88)	− 1.83 (5.65)	0.70 (6.81)	1.04 (11.19)	2.35 (11.69)	− 1.83 (29.45)
Levine et al. (2005)												
TBI (all types)	N/A	N/A	N/A	N/A	N/A	4.10 (20.40)	N/A	N/A	N/A	N/A	N/A	14.80 (21.40)
Minjoz et al. (2023)												
ABI	− 1.71 (5.22)	− 2.81 (4.75)	− 2.67 (7.86)	− 0.71 (7.38)	− 3.29 (7.29)	− 11.19 (20.35)	− 2.80 (3.35)	4.73 (7.62)	6.93 (7.14)	8.63 (8.34)	8.03 (9.25)	25.53 (22.14)
TBI (all types)	− 2.40 (8.17)	− 2.00 (10.30)	− 6.80 (12.85)	− 2.80 (13.08)	− 8.80 (10.06)	− 22.80 (49.02)	− 2.80 (3.35)	4.73 (7.62)	6.93 (7.14)	8.63 (8.34)	8.03 (9.25)	25.53 (22.14)
Ouerchefani et al. (2017)												
Focal frontal lesions	− 4.00 (4.71)	0.22 (7.99)	− 2.00 (7.27)	2.22 (10.26)	2.67 (9.38)	0.74 (17.77)	− 3.47 (4.98)	− 0.06 (6.18)	4.47 (6.44)	8.24 (8.82)	10.94 (5.70)	20.10 (13.86)
Robb and Good (2011)												
Mild TBI	− 2.40 (4.09)	− 2.00 (5.08)	1.00 (4.83)	− 0.35 (6.81)	0.17 (8.64)	− 3.57 (17.59)	− 2.17 (3.95)	0.87 (6.92)	1.13 (7.65)	1.20 (8.90)	1.40 (8.46)	2.43 (27.65)
Robb and Good (2014)												
Mild TBI	− 1.31 (5.89)	1.38 (7.51)	0.75 (7.29)	0.94 (8.41)	2.69 (7.07)	4.44 (24.26)	− 1.91 (8.11)	0.65 (8.94)	5.49 (8.17)	1.86 (8.26)	2.93 (10.01)	9.02 (27.45)
Moderate TBI	− 0.22 (5.33)	0.89 (6.57)	1.33 (4.36)	0.67 (8.12)	0.89 (6.33)	3.56 (16.09)	− 1.91 (8.11)	0.65 (8.94)	5.49 (8.17)	1.86 (8.26)	2.93 (10.01)	9.02 (27.45)
Scheffer et al. (2011)												
Stroke—female	− 0.07 (7.54)	− 4.66 (6.70)	− 3.55 (8.53)	10.49 (1.11)	10.19 (4.00)	− 14.00 (26.40)	− 0.44 (5.20)	3.44 (6.31)	7.44 (7.41)	6.00 (9.10)	5.89 (9.03)	23.00 (19.23)
Stroke—male	− 1.60 (3.50)	− 3.40 (8.05)	− 5.00 (4.34)	− 1.40 (4.71)	− 3.60 (8.83)	− 15.00 (19.39)	− 0.44 (5.20)	3.44 (6.31)	7.44 (7.41)	6.00 (9.10)	5.89 (9.03)	23.00 (19.23)
Sigurdardottir et al. (2010)												
Mild TBI	− 3.70 (4.70)	− 0.20 (6.30)	− 0.10 (9.30)	0.70 (9.10)	1.10 (8.80)	N/A	− 3.13 (6.81)	0.50 (6.42)	1.13 (9.68)	− 2.88 (11.26)	0.88 (8.45)	N/A
Moderate TBI	− 2.40 (5.90)	0.60 (5.00)	− 0.60 (7.10)	2.00 (8.20)	2.00 (8.30)	N/A	− 3.13 (6.81)	0.50 (6.42)	1.13 (9.68)	− 2.88 (11.26)	0.88 (8.45)	N/A
Severe TBI	− 2.30 (3.70)	− 2.20 (3.80)	− 1.20 (5.30)	− 1.60 (7.00)	− 1.80 (7.00)	N/A	− 3.13 (6.81)	0.50 (6.42)	1.13 (9.68)	− 2.88 (11.26)	0.88 (8.45)	N/A
Van Noordt et al. (2010)												
Mild TBI	− 3.78 (3.81)	1.67 (6.87)	6.44 (8.25)	4.00 (7.48)	5.33 (9.08)	13.66 (27.92)	− 4.15 (4.52)	1.08 (5.69)	3.23 (9.33)	2.62 (10.85)	3.15 (11.88)	5.93 (34.67)
Wang et al. (2021)												
DLPFC	N/A	N/A	N/A	N/A	N/A	2.93 (22.80)	N/A	N/A	N/A	N/A	N/A	11.44 (19.28)
VMPFC	N/A	N/A	N/A	N/A	N/A	−2.00 (17.59)	N/A	N/A	N/A	N/A	N/A	11.44 (19.28)
Xi et al. (2011)												
DLPFC	− 1.28 (4.47)	− 0.85 (4.55)	− 0.57 (2.65)	2.00 (4.43)	2.57 (5.57)	N/A	− 1.73 (3.77)	− 0.66 (4.01)	1.33 (4.52)	1.53 (4.62)	4.13 (4.60)	N/A
VMPFC	− 1.12 (3.34)	− 1.25 (4.78)	− 2.00 (2.30)	− 1.75 (4.18)	− 2.25 (5.45)	N/A	− 1.73 (3.77)	− 0.66 (4.01)	1.33 (4.52)	1.53 (4.62)	4.13 (4.60)	N/A
Zhang (2017)												
Mild TBI	N/A	N/A	N/A	N/A	N/A	7.96 (25.85)	N/A	N/A	N/A	N/A	N/A	21.27 (28.78)
Zinchenko and Enikolopova (2017)												
Right frontal tumors	− 1.67 (4.25)	2.67 (2.99)	2.33 (6.02)	3.33 (8.24)	3.67 (8.86)	10.33 (14.21)	− 2.38 (6.53)	7.05 (6.89)	5.62 (7.17)	7.52 (9.73)	9.05 (7.76)	26.86 (24.68)

Note. Mean net raw scores were calculated by deducting good deck selections from bad deck selections ([C + D]-[A + B])

*TBI* traumatic brain injury, *DLPFC* dorsolateral prefrontal cortex, *VMPFC* ventromedial prefrontal cortex, *SD* standard deviation

Of the 39 included samples, there was a total of 2188 participants (994 ABI and 1194 controls). Mean age for the ABI samples ranged from 19.00 to 64.10, whilst average education in years ranged from 7.30 to 16.98. The overall percentage of males across all ABI samples was 64%. Independent samples *t*-tests indicated that the ABI and control groups did not significantly differ in average age or average education, but they did vary in proportion of males. These study characteristics are presented in Table 3. Due to the large volume of data, general study information and demographics are presented in Table 1 whilst IGT scores are shown in Table 2.

**Table 3:** Characteristics of samples used in meta-analysis

Characteristic	ABI sample	Controls	*p*
	(*n* = 994)	(*n* = 1194)	
Average age (years)			.805
Mean	42.43	41.68	
Range	19.00–64.10	18.88–59.28	
SD	12.78	13.60	
Average education (years)			.389
Mean	11.87	12.38	
Range	7.30–16.98	8.15–16.40	
SD	2.42	2.01	
Sex			.006**
% male	64	50	

Note. This analysis was conducted after data cleaning but prior to imputation, which is detailed in the methods section

*SD*, average standard deviation across the samples;* p*, *p* value

^**^p < .01

### Meta-analyses

**Fig. 2:**
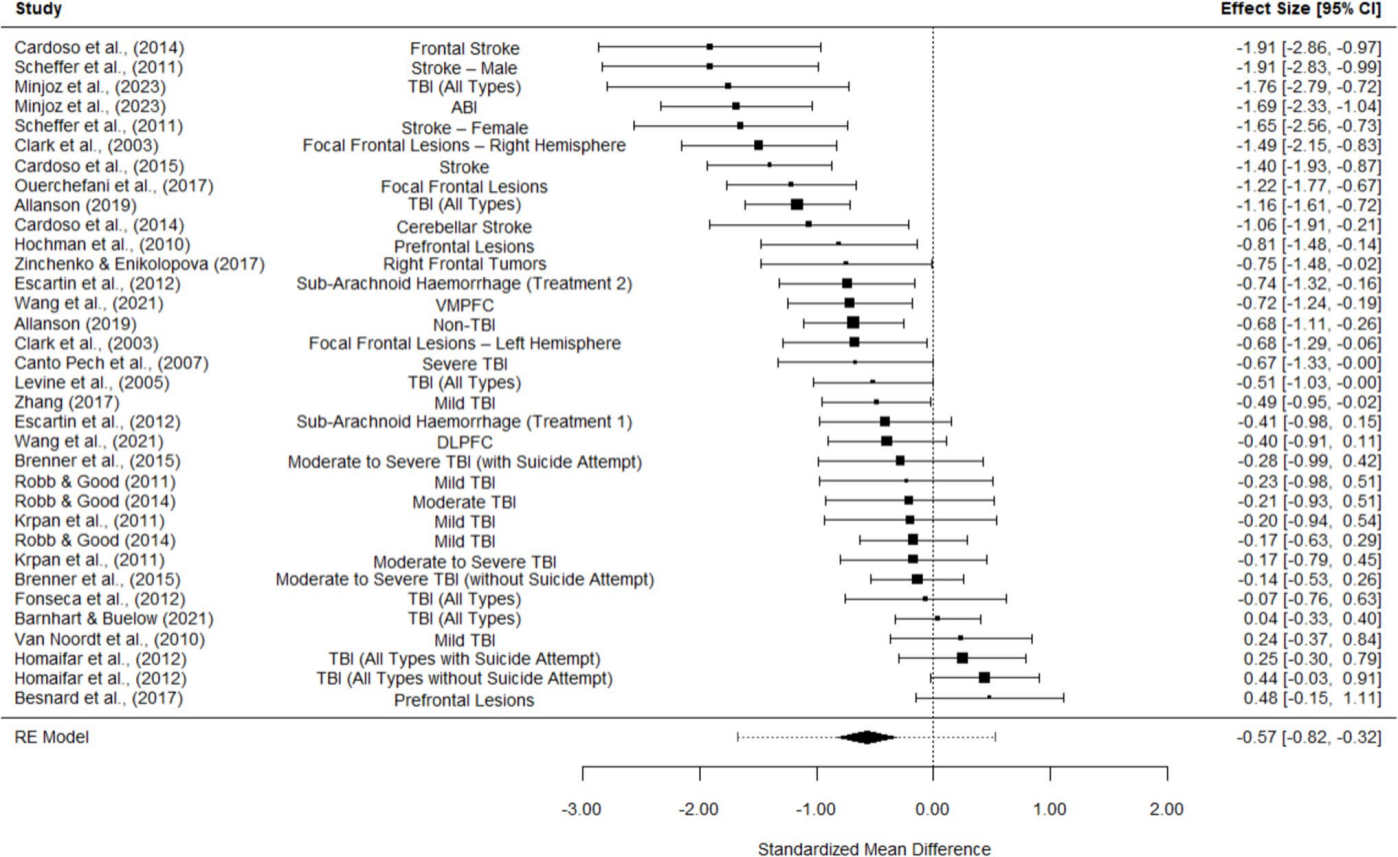
Forest plot for standardised mean difference (g) between clinical samples and controls on the IGT: total score. Note. Scores are calculated using mean net raw IGT scores ([C + D]-[A + B]). Pooled sample effect sizes are denoted by the black square; confidence intervals at 95% are represented by the horizontal line, whilst the size of the black square represents the sample weight. The dotted horizontal line denotes the pooled confidence intervals at 95%, and the black diamond represents the pooled effect size for all studies. IGT, Iowa Gambling Task; CI, confidence interval

Compared to controls, individuals with brain injury performed significantly worse on the IGT overall (*g* =  − 0.57, *p* < 0.001, 95% CI [− 0.82, − 0.32]; see Table 4). According to guidelines by Cohen (2013), this effect size can be regarded as moderate. The forest plot for this analysis is found in Fig. 2. In terms of block scores, no differences were found between clinical and control groups on block 1 (*g* = 0.01, *p* = 0.820, 95% CI [− 0.10, 0.13]). However, small to moderate statistically significant differences were found for block 2 (*g* =  − 0.28, *p* = 0.004, 95% CI [− 0.48, − 0.09]), block 3 (*g* =  − 0.49, *p* < 0.001, 95% CI [− 0.73, − 0.26]), block 4 (*g* =  − 0.31, *p* < 0.001, 95% CI [− 0.48, − 0.13]), and block 5 (*g* =  − 0.47, *p* < 0.001, 95% CI [− 0.66, − 0.28]). Cochrane’s *Q* was significant for all analyses excluding block 1. Heterogeneity (measured by *I*^2^) ranged from 53 to 78% across blocks 2 to totals—which is in the moderate to high range. Forest plots for block scores are found in the Figure S1.

**Table 4:** Meta-analyses of IGT blocks and total scores for ABI participants relative to controls

Section	*k*	*g*	*SE*	*p*	95% CI	Homogeneity statistics							
						*Q* (df)	*p*	Within study			Between study		
								*I*^2^	*σ*^2^	95% CI	*I*^2^	*τ*^2^	95% CI
Block 1	31	0.01	0.06	.820	[− 0.10, 0.13]	18.94 (30)	.941	0%	0.00	[0.00, 0.02]	0%	0.00	[0.00, 0.05]
Block 2	31	− 0.28	0.10	.004**	[− 0.48, − 0.09]	55.78 (30)	.003**	1%	0.00	[0.00, 0.08]	54%	0.12	[0.01, 0.31]
Block 3	31	− 0.49	0.12	< .001***	[− 0.73, − 0.26]	79.80 (30)	< .001***	3%	0.01	[0.00, 0.09]	66%	0.20	[0.06, 0.50]
Block 4	31	− 0.31	0.09	< .001***	[− 0.48, − 0.13]	68.70 (30)	< .001***	49%	0.09	[0.02, 0.25]	4%	0.01	[0.00, 0.17]
Block 5	31	− 0.47	0.1	< .001***	[− 0.66, − 0.28]	81.25 (30)	< .001***	48%	0.11	[0.02, 0.31]	13%	0.03	[0.00, 0.26]
Total	34	− 0.57	0.13	< .001***	[− 0.82, − 0.32]	120.86 (33)	< .001***	8%	0.03	[0.00, 0.15]	70%	0.27	[0.10, 0.63]

Note. *IGT*, Iowa Gambling Task;* k*, number of studies used in analysis; *g*, Hedges’ *g*; *SE*, standard error; *CI*, confidence interval; *p*, *p* value; *Q*, Cochran’s *Q*; *df*, degrees of freedom; *τ*^*2*^, tau squared; *σ*^*2*^, sigma squared; *I*^*2*^, test of heterogeneity

^**^p < .01

^***^p < .001

The combination of blocks 2–5 (*g* =  − 0.78, *p* < 0.001, 95% CI [− 1.12, − 0.44]) and the combination of blocks 3–5 (*g* =  − 0.74, *p* < 0.001, 95% CI [− 1.04, − 0.43]) both showed a medium effect for ABI vs controls. However, heterogeneity increased to the high category (83–85%). These results are presented in Table S2.

### Moderator Analysis

#### Subgroup Analysis

When examining effects by population type (i.e., non-TBI vs TBI), there were significant differences between the controls and both the non-TBI group (*g* =  − 0.93, *p* < 0.001, 95% CI [− 1.22, − 0.64]), and the TBI-confirmed group (*g* =  − 0.49, *p* = 0.002, 95% CI [− 0.80, − 0.18]), but not between the controls and the TBI-self-report group (*g* =  − 0.07, *p* = 0.742, 95% CI [− 0.46, 0.33]). Of note, non-TBI and TBI-confirmed were significantly different from one other, indicating that the two groups, whilst both impaired compared to controls, perform differently, with the non-TBI group demonstrating a larger effect size.

IGT version did not influence sensitivity to ABI, with both the original version (*g* =  − 0.38, *p* = 0.009, 95% CI [− 0.66, − 0.10]) and minor modifications (*g* =  − 0.94, *p* < 0.001, 95% CI [− 1.34, − 0.54]) showing impaired performance compared to controls. However, statistically significant differences between the original and minor modifications IGT were observed. Despite this, the sample that utilised the original sample was disproportionately made up of TBI groups, which were associated with smaller effect sizes.

Finally, when controlling for region, there were mixed findings. Significant differences on the IGT were found between clinical and non-clinical groups from the Asia Pacific (*g* =  − 0.74, *p* = 0.021, 95% CI [− 1.37, − 0.11]), Europe (*g* =  − 0.79, *p* < 0.001, 95% CI [− 1.17, − 0.41]), and Latin America (*g* =  − 1.17, *p* < 0.001, 95% CI [− 1.70, − 0.63]). However, individuals with ABI from North America performed the same as controls (*g* =  − 0.18, *p* = 0.258, 95% CI [− 0.48, 0.13]). Results for all subgroup analyses are reported in Table S3.

#### Meta-regression

Amongst the continuous moderators, the proportion of males (*b* = 0.21, *p* = 0.645, 95% CI [− 0.52, 0.83]), average age (*b* =  − 0.01, *p* = 0.320, 95% CI [− 0.03, 0.01]), and average education (*b* = 0.03, *p* = 0.823, 95% CI [− 0.08, 0.10]) were all nonsignificant (see Table S4).

### Outlier Analysis

No outliers were identified for blocks 2 and 4 and the total score. However, for blocks 1, 3, and 5, outliers were found. Once these extreme values were removed, the findings remained the same for all sections: block 1 (*g* =  − 0.04, *p* = 0.560, 95% CI [− 0.08, − 0.15]), block 3 (*g* =  − 0.48, *p* < 0.001, 95% CI [− 0.70, − 0.25]), and block 5 (*g* =  − 0.45, *p* < 0.001, 95% CI [− 0.64, − 0.27]). This suggests that the differences between clinical and control groups are robust to extreme values. The results of these analyses are found in Table S5.

### Publication and Small Sample Bias

For blocks 1–5, visual inspection of funnel plots indicated that most samples scattered symmetrically within the funnel, suggesting a low probability of publication bias. However, for the total scores, several studies fell outside the funnel plot. These funnel plots are found in Figure S2. PET analysis revealed a significant negative association between effect sizes and their standard errors (*b* =  − 2.48, *p* = 0.030, 95% CI [− 4.71, − 0.24]), indicating that smaller studies with larger standard errors reported larger effect sizes. Similarly, PEESE showed a significant negative relationship between effect sizes and their variances (*b* =  − 3.81, *p* = 0.025, 95% CI [− 7.12, − 0.49]), and after adjusting for this bias, the intercept in the PEESE model was not statistically significant (*b* =  − 0.20, *p* = 0.333, 95% CI [− 0.59, 0.20]), suggesting that the overall effect size is not significantly different from zero when accounting for publication bias.

## Discussion

This meta-analysis explored the level and nature of impairment of individuals with ABI on the IGT via two methods: The first compared the performance of adults with brain injury (not accounting for lesion location or severity) and controls. The second assessed the influence of IGT scoring approaches by combining later block scores on the IGT (e.g., those focused on decision-making under risk). Additionally, exploratory analyses were conducted to investigate potential performance moderators and their impact on IGT scores.

### General Findings

The findings from the first analysis suggest that adults with brain injury performed significantly worse than controls on the IGT for the total score—with a moderate effect size. Differences between groups become apparent in block 2, as controls acquire the rules of the IGT, whilst individuals with brain injury continue to make poorer deck selections throughout the remainder of the task.

As expected, the combination of later block scores resulted in numerically larger effect sizes between individuals with ABI and controls. In particular, the effect size for the combination of block scores 2, 3, 4, and 5 increased compared to the total score, although still fell within the moderate category. However, the confidence intervals from the total and combination scores were overlapping, suggesting the results from the two methods were not significantly different from one another.

Interestingly, we also found contrary evidence to the “deck B phenomenon” (Lee et al., 2020), as our review found more recent studies did not appear to have smaller effect sizes. Therefore, concerns that controls are performing poorer than originally reported does not seem to impact the IGTs sensitivity to impairments in decision-making (Steingroever et al., 2013).

### Performance Moderators

Whilst some performance moderators did influence IGT performance, the findings were mixed and, in some cases, unexpected. When controlling for population type, the non-TBI group and TBI-confirmed groups were found to have poorer performance on the IGT compared to controls. However, the non-TBI group performed significantly worse than TBI-confirmed, with large and medium effect sizes, respectively, and there were no differences found for the self-reported TBI group compared to controls. These results may seem counterintuitive, as histopathological and behavioral studies highlight the nature of traumatic injury involving multiple mechanisms with sustained injury cascades that can lead to long-term cognitive deficits (Bramlett & Dietrich, 2015). Of note, TBI often occurs in younger individuals with higher recovery rates compared to non-TBI conditions, whilst cardiovascular incidents are more common with advancing age (Colantonio et al., 2011; Cullen et al., 2008). Therefore, age differences between these subgroups may explain the finding of more impaired performance for non-TBI compared with the TBI groups. However, whilst MacPherson et al. (2009) found both TBI and stroke participants performed significantly poorer than controls on the IGT, there were no significant differences in performance between stroke and TBI participants. Given these mixed findings and limitations in the extant literature, further research is needed to better understand the role of injury type in IGT performance following ABI.

Moderator analyses also revealed that individuals who self-reported their TBI (versus TBI confirmed via clinical assessment/medical records) performed the same as controls—which was consistent with findings from Barnhart and Buelow (2021). Possible reasons to account for this finding include the following: the injury was so mild that it may not have resulted in dysexecutive symptoms, the patients may have recovered by the time they were assessed, or the methods of capturing brain injury may have been inadequate. Notably, self-reports of details regarding brain injury can be unreliable, which is unsurprising given the acute effects of TBI. For example, McKinlay et al. (2016) questioned a sample of 1003 people and found an overall accuracy of around 51% for reported TBI incidences. Therefore, whilst the present review included studies with self-reported TBI to ensure broad representation, it may be preferable for future studies to rely on medically documented TBI samples to ensure accurate characterisation of the injury.

Differences were found between the original and minor modification versions of the IGT, but both versions were sensitive to impaired performance compared to controls. This finding is somewhat surprising given the very minor nature of the modifications (i.e., language and currency adaptations). Although, as previously mentioned, this may be because the TBI group were primarily tested using the original version of the task. Therefore, it appears that this finding is an artifact of sampling and the modifications required for this test to be administered cross-culturally are unlikely to have a substantial effect on the task’s construct validity and sensitivity to brain injury.

A similar pattern of findings appears to explain differences across regions, with all regions demonstrating impaired performance on the IGT bar North America. However, most of the TBI samples fell into this category, which may account for the lack of significance. This suggests that there are no differences in ABI performance on the IGT regardless of geographic location, which is consistent with findings by Lee et al. (2020).

In terms of demographics, average education, average age, and proportion of males did not significantly predict IGT performance in individuals with brain injury. Whilst the relationship between age and IGT performance in the previous literature is somewhat inconsistent (Beitz et al., 2014; Denburg et al., 2005; Wood et al., 2005), the few studies that have specifically examined individuals with brain injury found no relationship between age and IGT scores (MacPherson et al., 2009; Scheffer et al., 2011). So, whilst age-related performance was deemed relevant enough that age-corrected norms were included in the IGT PAR manual, our findings indicate that age does not significantly predict IGT performance. In terms of gender, our findings are consistent with other findings with ABI samples (MacPherson et al., 2009; Scheffer et al., 2011).

### Strengths and Limitations

The comprehensive approach of this study is a key strength. Specifically, the evaluation of multiple performance indicators of the IGT (i.e., total score vs combination block scores), as well as the inclusion of a large enough sample of papers, allowed for several important moderating factors within this review, which to our knowledge has not been conducted for this cohort thus far. However, in the process of doing so, we identified several important limitations within the IGT ABI literature. Many studies, as is typical when examining individuals with brain injury, had small sample sizes (*n* = 5–99). In addition, 29 relevant studies that met criteria during the search process were excluded due to inaccessible data. Of the studies that were included, many were missing data required for certain analyses—such as block/total scores or demographic data. Furthermore, whilst it was a goal to investigate aspects such as lesion location or time since injury, this analysis was not feasible due to the lack of available data. As such, whilst these findings provide support regarding the sensitivity of the IGT to detect impairment in brain injury, there is insufficient evidence regarding the IGT’s ability to detect impairment relative to specific brain regions.

The heterogenous nature of ABI is another limitation in interpreting study findings. Not only are there important differences in the mechanisms, lesion location, severity, symptoms, treatment options, and recovery trajectories for both non-TBI and TBI conditions (Cullen et al., 2008; Zhang et al., 2016), but there are variations within these groups (Morgan & Ricker, 2018). As previously mentioned, the influence of average age on IGT performance may have been impacted by group differences between non-TBI and TBI samples within this paper. Additionally, it is likely that the nature of the methodological approach taken by the studies also impacted the findings. Specifically, most studies looking at IGT performance in TBI were characterised by generally mild to moderate levels of injury with few severe cases. Therefore, the finding that the TBI-confirmed group had a smaller effect size than the non-TBI group, and that the self-report TBI group performed the same as controls, may be a consequence of limited sampling, as opposed to an accurate representation of the level and nature of impairment on the IGT in TBI in more severe cases.

The results of this study do not speak to the ecological validity of the task—that is, the relationship between task performance and real-world risky decision-making. There is limited evidence in the research that has explored this aspect following brain injury (e.g., Levine et al., 2005), with the research skewing towards other clinical populations such as substance use (Domínguez-Salas et al., 2016; Passetti et al., 2008; Verdejo-Garcia et al., 2006). Therefore, whilst there is some data to suggest the IGT’s relevance in predicting outcomes for individuals with ABI, further exploration is needed to fully understand its ecological validity.

Finally, we acknowledge that the retest reliability of the Iowa Gambling Task (IGT) is low to moderate, which may lead to unwarranted construct proliferation and attenuated validity correlations (Schmidt & Hunter, 1996). Consequently, these reliability concerns may explain some aspects of our findings.

### Implications and Directions for Future Research

This research provides a significant contribution to the existing IGT literature, as it is the first meta-analysis to focus on an ABI population. The findings have both theoretical and practical applications. In particular, whilst this study suggests that hot EF (as measured by the IGT) appears to be sensitive to ABI, clinicians should carefully consider its psychometric limitations before including it in a neuropsychological test battery. Whether it adds additional information in the context of cold EF remains unknown, although some preliminary evidence suggests that that may be the case (Chavez-Arana et al., 2018). Further research is needed in this area, especially in terms of the conceptualisation of hot EF. Salehinejad et al. (2021) stress the importance of thinking about EF as a complex interrelated system, and therefore, damage to one region can have cascading impacts on other regions. Therefore, the inclusion of hot EF measures in neuropsychological test batteries may be beneficial, even when deficits in this area are not expected.

Furthermore, treatment recommendations vary based on deficits in hot vs cold domains (Chavez-Arana et al., 2018)—therefore, cognitive assessments that do not include hot EF measures may be missing opportunities to provide a more complementary treatment program. A more comprehensive EF battery will allow clinicians to have a greater understanding of an individuals’ cognitive profile and the challenges experienced. Individuals who are misdiagnosed are more likely to have a more challenging recovery, as they are less likely to have access to the resources and information that will allow them to make sense of their injury and associated symptoms (Prince & Bruhns, 2017).

As discussed, a relatively large percentage of these studies either had methodological limitations and/or small sample sizes. It certainly should be acknowledged that research in ABI is challenging. However, this remains a limitation that must be addressed in future research to enhance the design and implementation of studies in this population. For example, guidelines about what data to collect and report in ABI literature would greatly improve the consistency of injury classification and severity. Whilst classification systems such as the Glasgow Coma Scale (GCS) are useful in assessing severity in brain regions where medical imaging is not available, it can make it challenging to compare patients in both clinical practice and research (Carter et al., 2016). Alternative measures such as those proposed by Wilson et al. (2022), have been suggested to address these concerns. Future studies utilising the IGT are encouraged to provide as much information as possible such as net block and total scores. Based on the current meta-analysis, conclusions regarding the level of IGT impairment in individuals with more severe TBI are uncertain.

## Conclusion

This meta-analysis explored the level and nature of impairment of individuals with ABI on the IGT compared to controls. Overall, these findings support the notion that the IGT is sensitive to the presence of brain injury, particularly non-TBI and medically confirmed TBI, with differences becoming apparent in block 2. No differences were found between the use of combination of later block scores vs total IGT scores. Performance moderators such as population type and region influenced IGT performance, whilst average age, average education, and proportion of males did not. These findings suggest that the IGT is sensitive to impairments in decision-making amongst individuals with ABI.

## Supplementary Information

Below is the link to the electronic supplementary material.

Supplementary file1 (DOCX 1013 KB)

## Data Availability

The data set from this study are openly available on Open Science Framework which can be accessed via https://osf.io/ndheq/?view_only=d159935118bd4e33a5a8f5dc38764f58.

## Footnotes

1 The original search criteria only included studies published in English and populations from 18 to 70 years old. This was amended in PROSPERO on 29 November 2021 to allow for more studies for consideration.

2 These values were originally treated as SEM; however, when they were transformed into SD, the values seemed unrealistically large. For example, a SEM of 9.57 for control age resulted in a SD of 52.42. The authors of the paper were contacted to confirm these values, but no response was received.

3 The author of the paper was contacted to inform them of this finding.

4 Sensitivity analysis specifying the full sample size for this study was conducted and revealed no noticeable differences to either effect size or pooled effect size values.

5 There were no self-report non-TBI participants.

6 We provided the results for the imputed and non-imputed values in the supplementary materials (Tables S4 and S8, respectively).

7 The R code and data set used for these analyses are available online via https://osf.io/ndheq/.

8 Please see Figure S3 in the supplementary materials for formulas.

9 We wish to note that the PET-PEESE approach was developed specifically for univariate analyses, and its application to multivariate analyses should be interpreted cautiously.

10 Krpan control data came from Krpan, K. M., Stuss, D. T., and Anderson, N. D. (2011b). Planful versus avoidant coping: Behavior of individuals with moderate-to-severe traumatic brain injury during a psychosocial stress test. *Journal of the International Neuropsychological Society*, *17*(2), 248–255. https://doi.org/10.1017/s1355617710001499
