# Supplementary material for: The Level and Nature of Impairment on the Iowa Gambling Task Following Acquired Brain Injury: A Meta-analysis
Source: Neuropsychol Rev. 2025 Jun 14;36(2):140–59. doi: 10.1007/s11065-025-09668-4 (PMC13388515; doi:10.1007/s11065-025-09668-4)
Supplement: Supplementary file 1 — Supplementary file1 (DOCX 1013 KB) [file 11065_2025_9668_MOESM1_ESM.docx]

Supplementary Materials

Table S1

Risk of Bias Quality Assessment Criteria Using QUADAS-2 Guidelines

|  | **Allanson (2019)** | **Barnhart & Buelow (2021)** | **Besnard et al., (2017)** | **Brenner et al., (2015)** | **Canto Pech et al., (2007)** | **Cardoso et al., (2015)** | **Cardoso et al., (2014)** | **Clark et al., (2003)** | **Escartin et al., (2012)** | **Fonseca et al., (2012)** | **Hochman et al., (2010)** | **Homaifar et al., (2012)** | **Krpan et al., (2011)** | **Levine et al., (2005)** | **Ouerchefani et al., (2017)** | **Robb & Good (2011)** | **Robb & Good (2014)** | **Scheffer et al., (2011)** | **Sigurdardottir et al., (2010)** | **Van Noordt et al., (2010)** | **Wang et al., (2021)** | **Xi et al., (2011)** | **Zhang (2017)** | **Zinchenko & Enikolopova (2017)** | **Minjoz et al., (2023)** |
| --- | --- | --- | --- | --- | --- | --- | --- | --- | --- | --- | --- | --- | --- | --- | --- | --- | --- | --- | --- | --- | --- | --- | --- | --- | --- |
| **DOMAIN 1: PATIENT SELECTION** |  |  |  |  |  |  |  |  |  |  |  |  |  |  |  |  |  |  |  |  |  |  |  |  |  |
| **A. Risk of Bias - Assess the risk of bias associated with the way participants were selected for the study.** |  |  |  |  |  |  |  |  |  |  |  |  |  |  |  |  |  |  |  |  |  |  |  |  |  |
| Was a consecutive sample of patients enrolled during a specified period? | Y | Y | Y | Y | Y | Y | Y | U | Y | Y | Y | U | Y | Y | Y | Y | Y | Y | Y | Y | Y | Y | Y | Y | Y |
| Did the study avoid inappropriate exclusions? | Y | Y | Y | Y | Y | Y | Y | Y | Y | Y | Y | Y | Y | Y | Y | Y | Y | Y | Y | Y | Y | Y | Y | Y | Y |
| Could the selection of patients have introduced bias? | L | L | L | L | L | L | L | L | L | L | L | L | L | L | L | L | L | L | L | L | L | L | L | L | L |
| **B. Concerns regarding applicability** |  |  |  |  |  |  |  |  |  |  |  |  |  |  |  |  |  |  |  |  |  |  |  |  |  |
| Is there concern that the included patients do not match the review question? | L | L | L | L | L | L | L | L | L | L | L | L | L | L | L | L | L | L | L | L | L | L | L | L | L |
| **DOMAIN 2: INDEX TEST (IOWA GAMBLING TASK)** |  |  |  |  |  |  |  |  |  |  |  |  |  |  |  |  |  |  |  |  |  |  |  |  |  |
| **A. Risk of Bias - Evaluate the risk of bias in the conduct and interpretation of the index test (IGT).** |  |  |  |  |  |  |  |  |  |  |  |  |  |  |  |  |  |  |  |  |  |  |  |  |  |
| Was the Iowa Gambling Task (IGT) administered and interpreted in a standardized manner for all participants? | Y | Y | Y | Y | Y | Y | Y | Y | Y | Y | Y | Y | Y | Y | Y | Y | Y | Y | Y | Y | Y | Y | Y | Y | Y |
| Could the conduct or interpretation of the index test have introduced bias? | L | L | L | L | L | L | L | L | L | L | L | L | L | L | L | L | L | L | L | L | L | L | L | L | L |
| **B. Concerns regarding applicability** |  |  |  |  |  |  |  |  |  |  |  |  |  |  |  |  |  |  |  |  |  |  |  |  |  |
| Is there concern that the index test, its conduct, or interpretation differ from the review question? | L | L | L | L | L | L | L | L | L | L | L | L | L | L | L | L | L | L | L | L | L | L | L | L | L |
| **DOMAIN 3: REFERENCE STANDARD (DIAGNOSIS OF ABI)** |  |  |  |  |  |  |  |  |  |  |  |  |  |  |  |  |  |  |  |  |  |  |  |  |  |
| **A. Risk of Bias - Assess the risk of bias in the reference standard used to diagnose acquired brain injury.** |  |  |  |  |  |  |  |  |  |  |  |  |  |  |  |  |  |  |  |  |  |  |  |  |  |
| Was the reference standard (diagnosis of ABI) valid, reliable, and applied equally to all participants? | Y | Y | Y | Y | Y | Y | Y | Y | Y | Y | Y | Y | Y | Y | Y | Y | Y | Y | Y | Y | Y | Y | Y | Y | Y |
| Were the interpreters of the IGT results blinded to the participants' clinical diagnosis or severity of brain injury? | N | N | N | N | N | N | N | N | N | N | N | N | N | N | N | N | N | N | N | N | N | N | N | N | N |
| Could the selection of patients have introduced bias? | L | L | L | L | L | L | L | L | L | L | L | L | L | L | L | L | L | L | L | L | L | L | L | L | L |
| **B. Concerns regarding applicability** |  |  |  |  |  |  |  |  |  |  |  |  |  |  |  |  |  |  |  |  |  |  |  |  |  |
| Is there concern that the target condition as defined by the reference standard does not match the review question? | L | L | L | L | L | L | L | L | L | L | L | L | L | L | L | L | L | L | L | L | L | L | L | L | L |
| **DOMAIN 4: FLOW AND TIMING** |  |  |  |  |  |  |  |  |  |  |  |  |  |  |  |  |  |  |  |  |  |  |  |  |  |
| **A. Risk of Bias - Evaluate whether there was appropriate timing and flow of participants through the study.** |  |  |  |  |  |  |  |  |  |  |  |  |  |  |  |  |  |  |  |  |  |  |  |  |  |
| Was the interval between the IGT (index test) and the ABI diagnosis (reference standard) appropriate and similar across all participants (i.e., acute [<2yrs] or post-acute)? | Y | U | Y | U | Y | Y | Y | U | Y | Y | Y | Y | U | Y | Y | U | Y | Y | Y | U | U | Y | U | Y | U |
| Did all participants receive the same index test and reference standard? | Y | Y | Y | Y | Y | Y | Y | Y | Y | Y | Y | Y | Y | Y | Y | Y | Y | Y | Y | Y | Y | Y | Y | Y | Y |
| Were all enrolled participants included in the analysis where feasible? | Y | Y | Y | Y | Y | Y | Y | Y | Y | Y | Y | Y | Y | Y | Y | Y | Y | Y | Y | Y | Y | Y | Y | Y | Y |
| Could the patient flow have introduced bias? | L | L | L | L | L | L | L | L | L | L | L | L | L | L | L | L | L | L | L | L | L | L | L | L | L |

*Note.* Y = Yes, N = No, L = Low, U = Unclear.

Table S2

Between Groups Meta-Analytic Results: Combination of Blocks 2-5 and 3-5 and Total Scores

| Section | *k* | *g* | *SE* | *p* | *95% CI* | Homogeneity statistics | | | | | | | |
| --- | --- | --- | --- | --- | --- | --- | --- | --- | --- | --- | --- | --- | --- |
|  |  |  |  |  |  | *Q (df)* | *p* | Within study | | | Between study | | |
|  |  |  |  |  |  |  |  | I² | *σ²* | 95% CI | I² | *τ²* | 95% CI |
| Total | 34 | -0.57 | 0.13 | < .001*** | [-0.82, -0.32] | 120.86 (33) | < .001*** | 8% | 0.03 | [0.00, 0.15] | 70% | 0.27 | [0.10, 0.63] |
| Blocks 2-5 | 31 | -0.78 | 0.17 | < .001*** | [-1.12, -0.44] | 171.77 (30) | < .001*** | 28% | 0.18 | [0.05, 0.58] | 57% | 0.37 | [0.01, 1.01] |
| Blocks 3-5 | 31 | -0.74 | 0.15 | < .001*** | [-1.04, -0.43] | 158.61 (30) | < .001*** | 46% | 0.25 | [0.08, 0.71] | 37% | 0.20 | [0.00, 0.72] |

*Note.* Total score included for comparison purposes. *k* = number of studies used in analysis, g = Hedges’ g, *SE* = standard error, *p* = p value, *CI* = confidence interval, *Q* = Cochran's Q, *df* = degrees of freedom, *I*² = test of heterogeneity, *τ²* = tau squared*, σ²* = sigma squared.

Figure S1

Forest Plots for Standardised Mean Difference (g) Between Clinical Samples and Healthy Controls on the Iowa Gambling Task: Blocks 1-5

**Block 1**


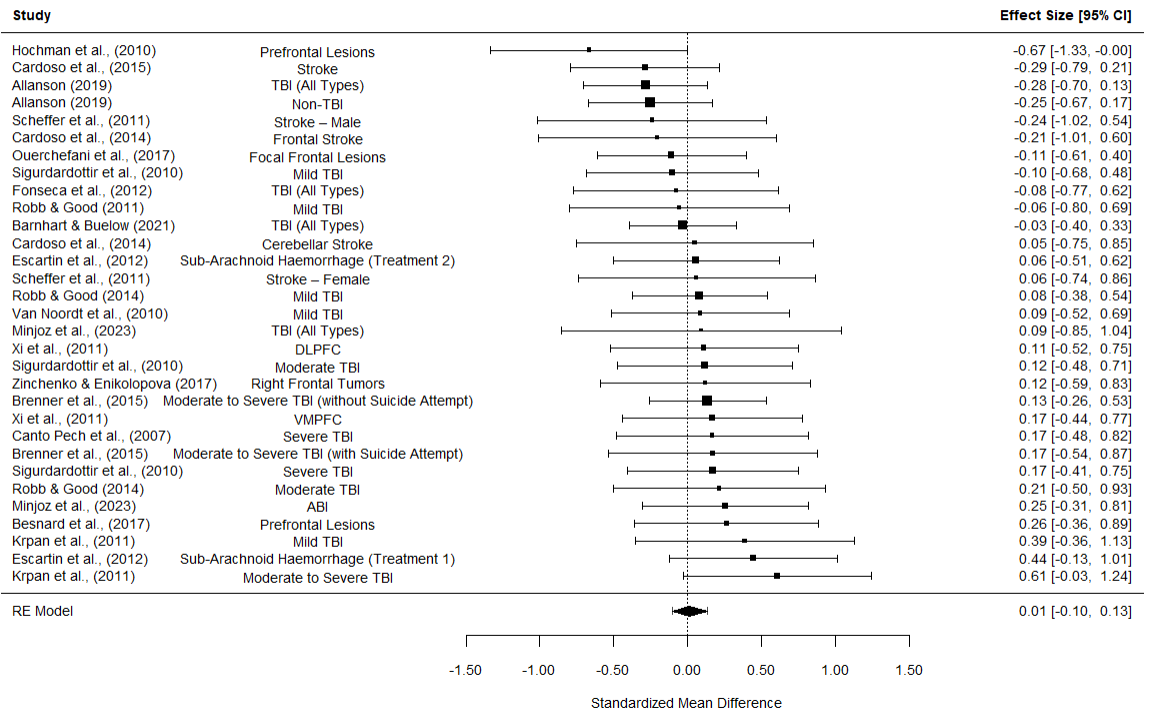


**Block 2**


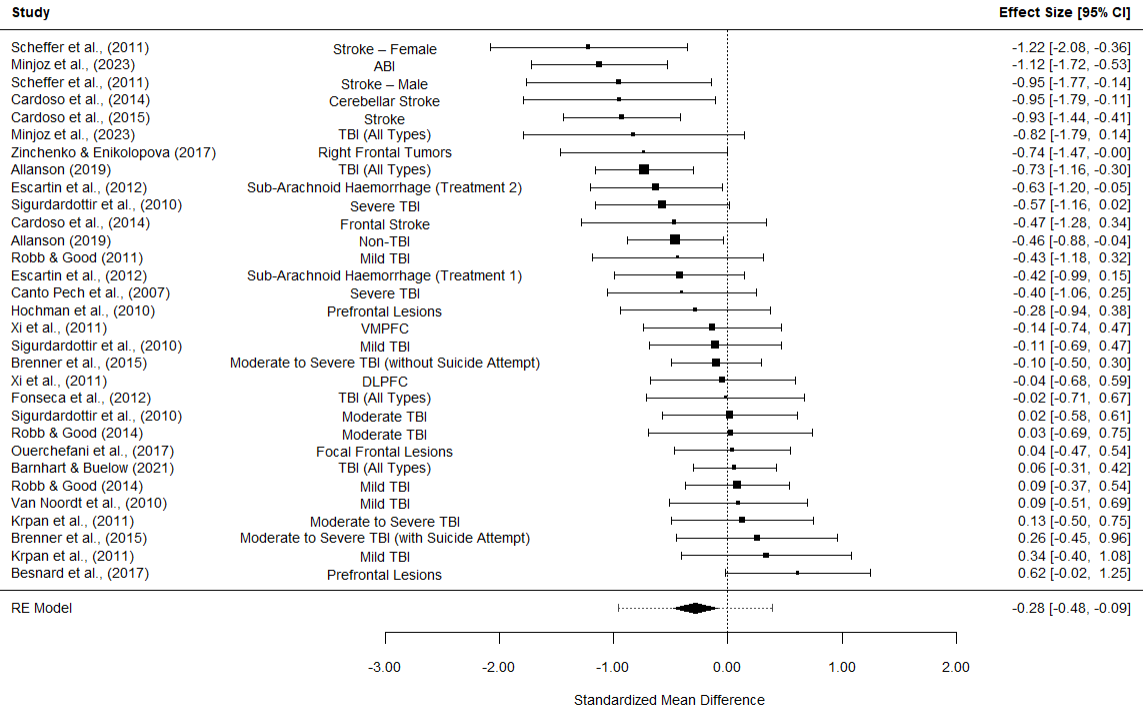


**Block 3**


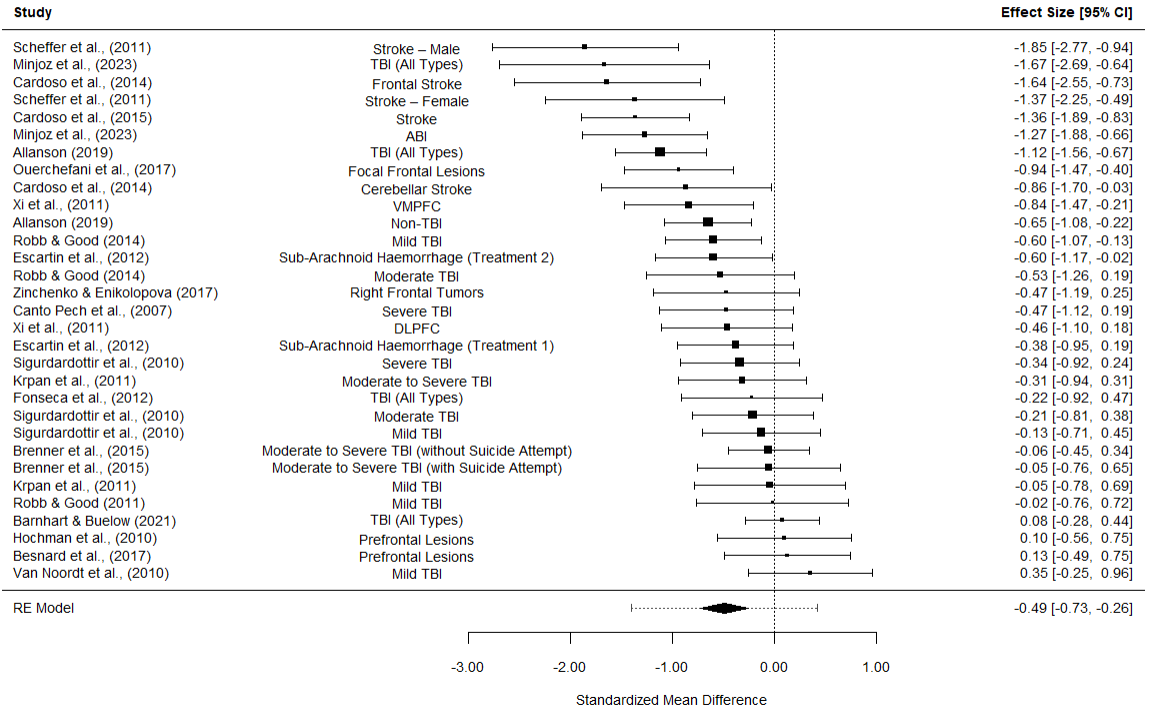


**Block 4**


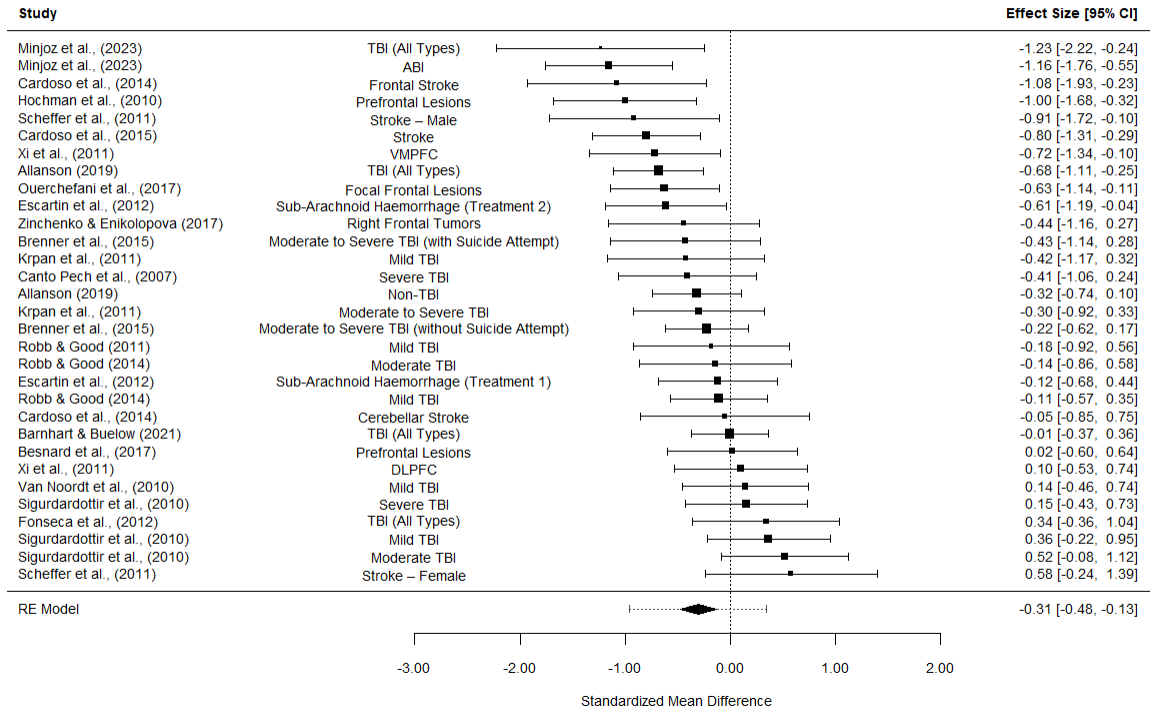


**Block 5**


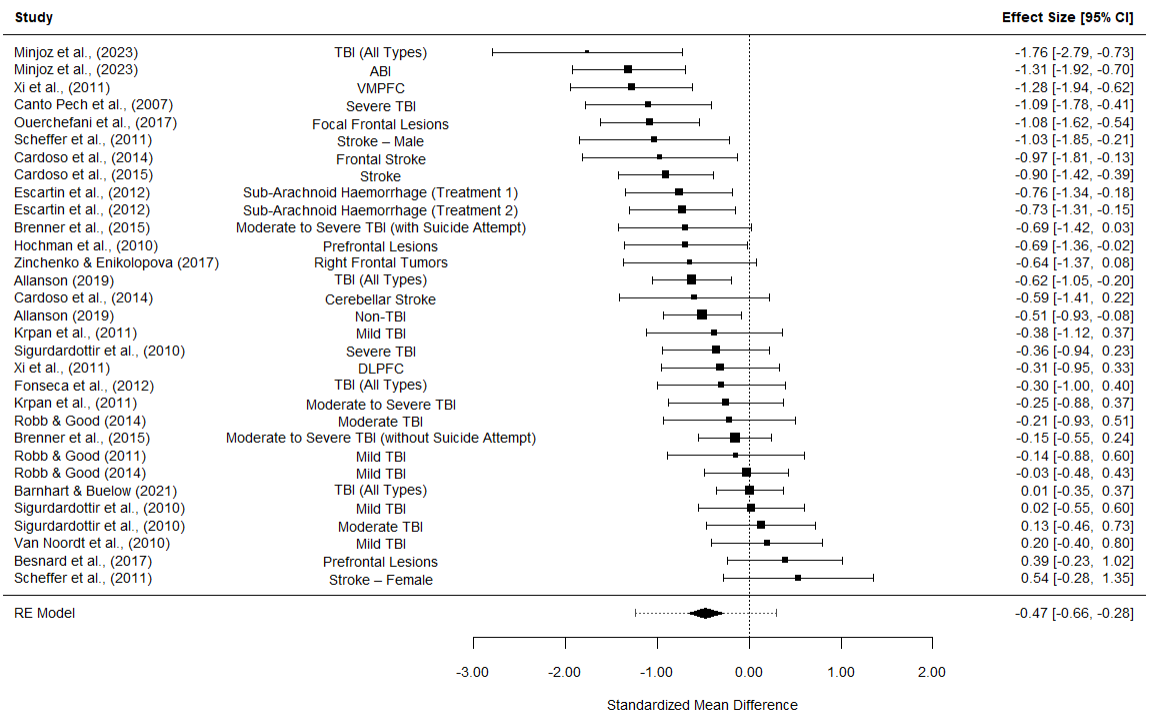


*Note.* Scores are calculated using mean net raw IGT scores ([C+D]-[A+B]). Pooled sample effect sizes are denoted by the black square, confidence intervals at 95% are represented by the horizontal line, whilst the size of the black square represents the sample weight. The dotted horizontal line denotes the pooled confidence intervals at 95%, and the black diamond represents the pooled effect size for all studies. IGT = Iowa Gambling Task, CI = confidence interval.

Table S3

Subgroup Analyses of Total Scores

| Section | *k* | *g* | *SE* | *p* | *95% CI* | Homogeneity statistics | |  |
| --- | --- | --- | --- | --- | --- | --- | --- | --- |
|  |  |  |  |  |  | *QE (df)* | *p* |  |
|  |  |  |  |  |  |  |  |  |
| Population | 31 |  |  |  |  | 73.24 (28) | < .001*** |  |
| Non TBI | 14 | -0.93 | 0.15 | < .001*** | [-1.22, -0.64] |  |  |  |
| TBI ‒ Confirmed | 10 | -0.49 | 0.16 | 0.002** | [-0.80, -0.18] |  |  |  |
| TBI ‒ Self-Report | 7 | -0.07 | 0.20 | 0.742 | [-0.46, 0.33] |  |  |  |
| IGT Version | 34 |  |  |  |  | 101.09 (32) | < .001*** |  |
| Original | 22 | -0.38 | 0.14 | 0.009** | [-0.66, -0.10] |  |  |  |
| Minor Modifications | 12 | -0.94 | 0.20 | < .001*** | [-1.34, -0.54] |  |  |  |
| Region | 34 |  |  |  |  | 75.76 (30) | < .001*** |  |
| North America | 14 | -0.18 | 0.16 | 0.258 | [-0.48, 0.13] |  |  |  |
| Asia Pacific | 4 | -0.74 | 0.32 | 0.021* | [-1.37, -0.11] |  |  |  |
| Europe | 10 | -0.79 | 0.19 | < .001*** | [-1.17, -0.41 |  |  |  |
| Latin America | 6 | -1.17 | 0.27 | < .001*** | [-1.70, -0.63 |  |  |  |

*Note.* Original IGT includes both in person and computer versions of the task. IGT = Iowa Gambling Task, TBI = Traumatic Brain Injury, *k* = number of studies used in analysis, *b* = regression coefficient, g = Hedges’ g, *SE* = standard error, *CI* = confidence interval, *p* = p value, *I*² = test of heterogeneity, *QE* = residual heterogeneity, *df* = degrees of freedom.

* *p* < .05

****p* < .001

Table S4

Meta Regression of Total Scores

| Model | *k* | *b* | *SE* | *p* | *95% CI* | Homogeneity statistics | |  |
| --- | --- | --- | --- | --- | --- | --- | --- | --- |
|  |  |  |  |  |  | *QE (df)* | *p* |  |
|  |  |  |  |  |  |  |  |  |
| % Male | 34 | 0.21 | 0.36 | 0.645 | [-0.52, 0.83] | 111.32 (30) | < .001*** |  |
| Average Age | 34 | -0.01 | 0.01 | 0.32 | [-0.03, 0.01] | 111.32 (30) | < .001*** |  |
| Average Education | 34 | 0.03 | 0.05 | 0.823 | [-0.08, 0.10] | 111.32 (30) | < .001*** |  |

*Note.* *k* = number of studies used in analysis, *b* = regression coefficient, *SE* = standard error, *p* = p value, *CI* = confidence interval, *QE* = QE statistic, *df* = degrees of freedom. For the results of this analysis without imputed values please see table S8.

****p* < .001

Table S5

Outlier Analyses of Block and Total Scores

| Section | *k* | *g* | *SE* | *p* | *95% CI* | Homogeneity statistics | | | | | | | |
| --- | --- | --- | --- | --- | --- | --- | --- | --- | --- | --- | --- | --- | --- |
|  |  |  |  |  |  | *Q (df)* | *p* | Within study | | | Between study | | |
|  |  |  |  |  |  |  |  | I² | *σ²* | 95% CI | I² | *τ²* | 95% CI |
| Block 1 | 31 | 0.01 | 0.06 | 0.820 | [-0.10, -0.13] | 18.94 (30) | 0.941 | 0% | 0.00 | [0.00, 0.02] | 0% | 0.00 | [0.00, 0.05] |
| Block 1^a^ | 30 | 0.04 | 0.06 | 0.560 | [-0.08, -0.15] | 14.81 (29) | 0.987 | 0% | 0.00 | [0.00, 0.02] | 0% | 0.00 | [0.00, 0.04] |
| Block 2 | 31 | -0.28 | 0.10 | 0.004** | [-0.48, -0.09] | 55.78 (30) | 0.003** | 1% | 0.00 | [0.00, 0.08] | 54% | 0.12 | [0.01, 0.31] |
| Block 3 | 31 | -0.49 | 0.12 | < .001*** | [-0.73, -0.26] | 79.80 (30) | < .001*** | 3% | 0.01 | [0.00, 0.09] | 66% | 0.20 | [0.06, 0.50] |
| Block 3^b^ | 30 | -0.48 | 0.12 | < .001*** | [-0.70, -0.25] | 74.53 (29) | < .001*** | 3% | 0.01 | [0.00, 0.10] | 64% | 0.17 | [0.04, 0.46] |
| Block 4 | 31 | -0.31 | 0.09 | < .001*** | [-0.48, -0.13] | 68.70 (30) | < .001*** | 49% | 0.09 | [0.02, 0.25] | 4% | 0.01 | [0.00, 0.17] |
| Block 5 | 31 | -0.47 | 0.1 | < .001*** | [-0.66, -0.28] | 81.25 (30) | < .001*** | 48% | 0.11 | [0.02, 0.31] | 13% | 0.03 | [0.00, 0.26] |
| Block 5^c^ | 30 | -0.45 | 0.09 | < .001*** | [-0.64, -0.27] | 79.58 (29) | < .001*** | 61% | 0.14 | [0.03, 0.31] | 0% | 0.00 | [0.00, 0.20] |
| Total | 34 | -0.57 | 0.13 | < .001*** | [-0.82, -0.32] | 120.86 (33) | < .001*** | 8% | 0.03 | [0.00, 0.15] | 70% | 0.27 | [0.10, 0.63] |

*Note.* Letter superscripts denote the outlier analyses, with the original analyses for comparison. No outliers were found for Block 2, 4 and Total. *k* = number of studies used in analysis, *g* = Hedges’ g, *SE* = standard error, *CI* = confidence interval, *p* = p value, *I*² = test of heterogeneity, *Q* = Cochran’s Q, *df* = degrees of freedom, *τ²* = tau squared*, σ²* = sigma squared.

^a^Removed as outliers: Hochman et al., (2010)

^b^Removed as outliers: Scheffer et al., (2011) – Stroke − Male

^c^Removed as outliers: Minjoz et al., (2023) – ABI

* *p* < .05

***p* < .01

****p* < .001

Figure S2

Funnel Plots of Block and Total Scores


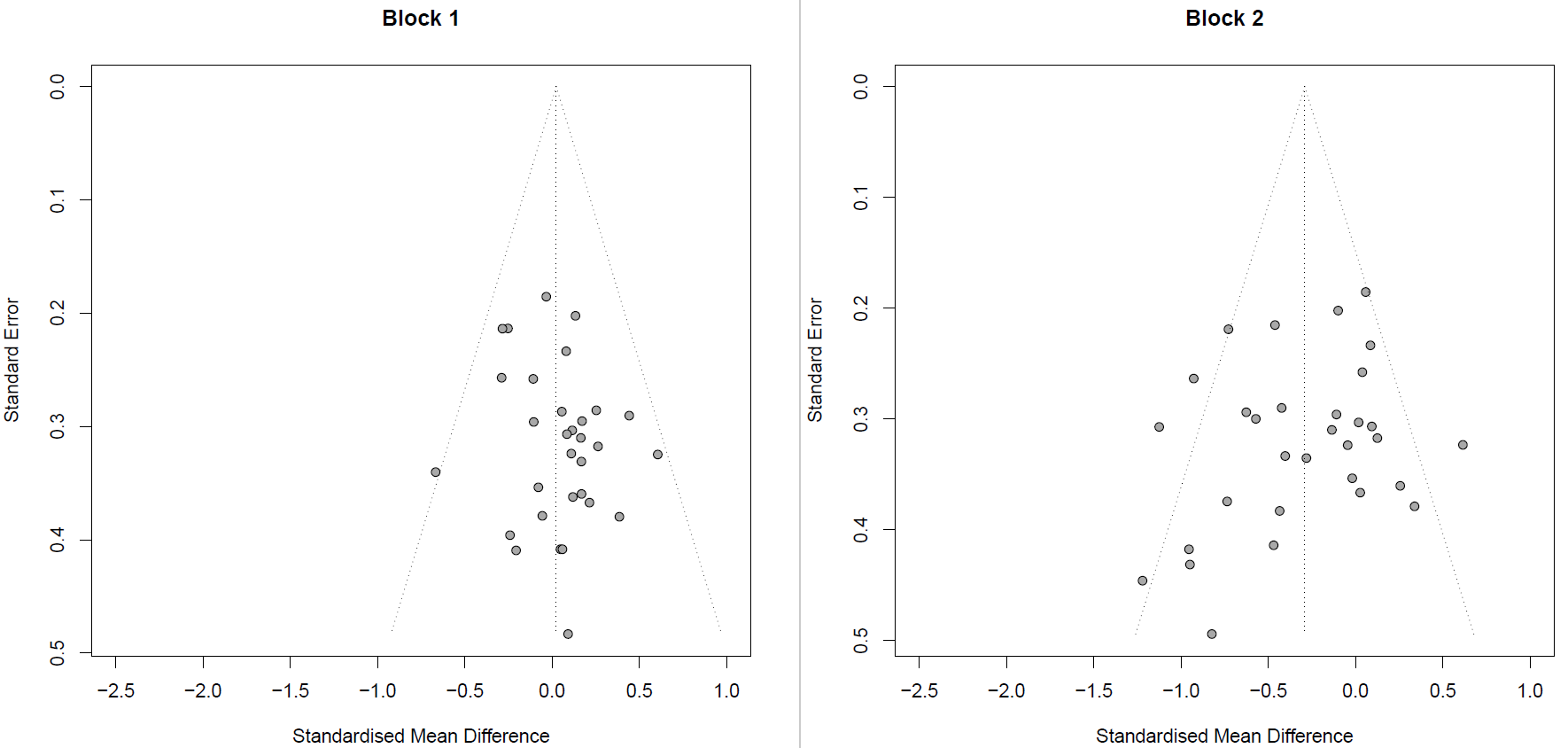


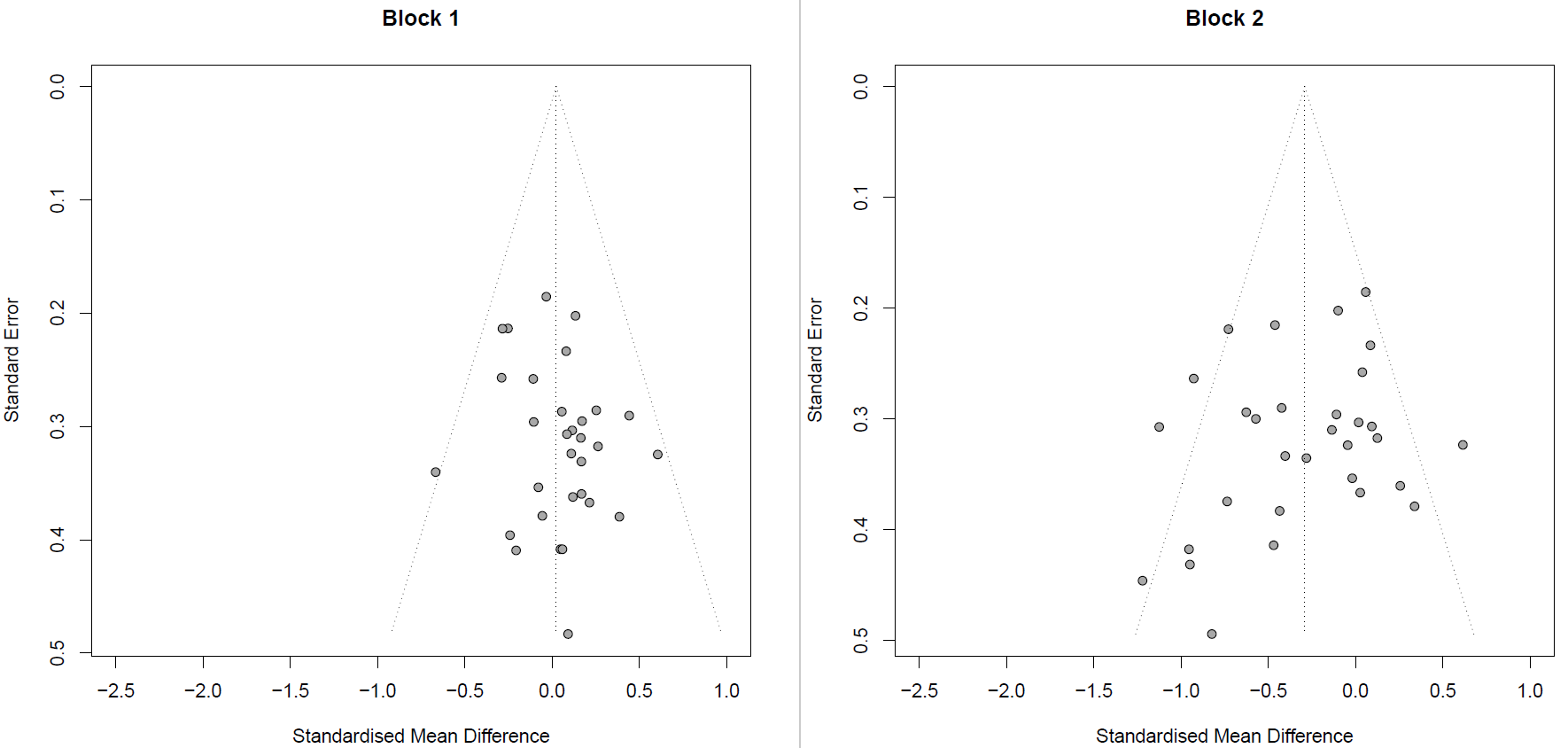


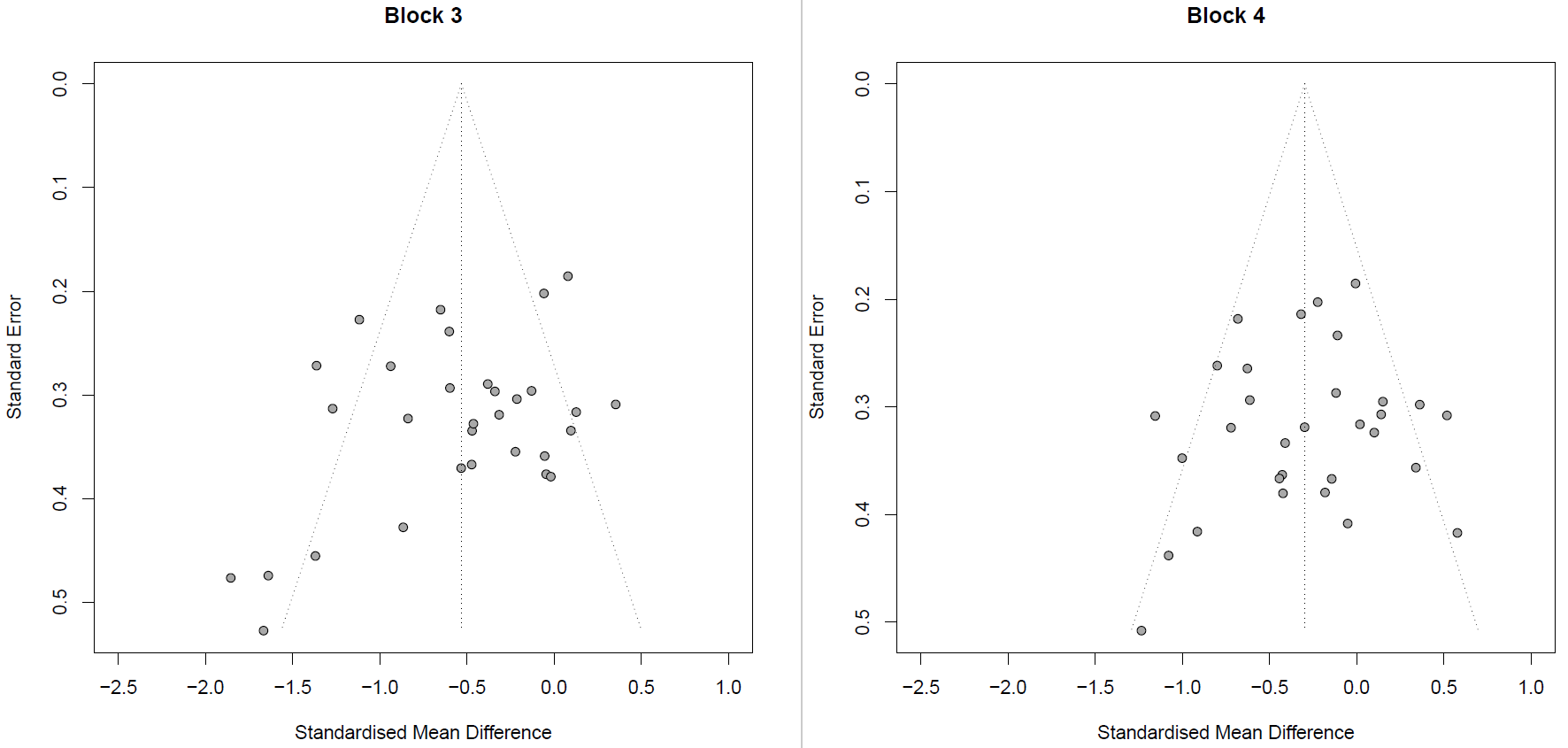


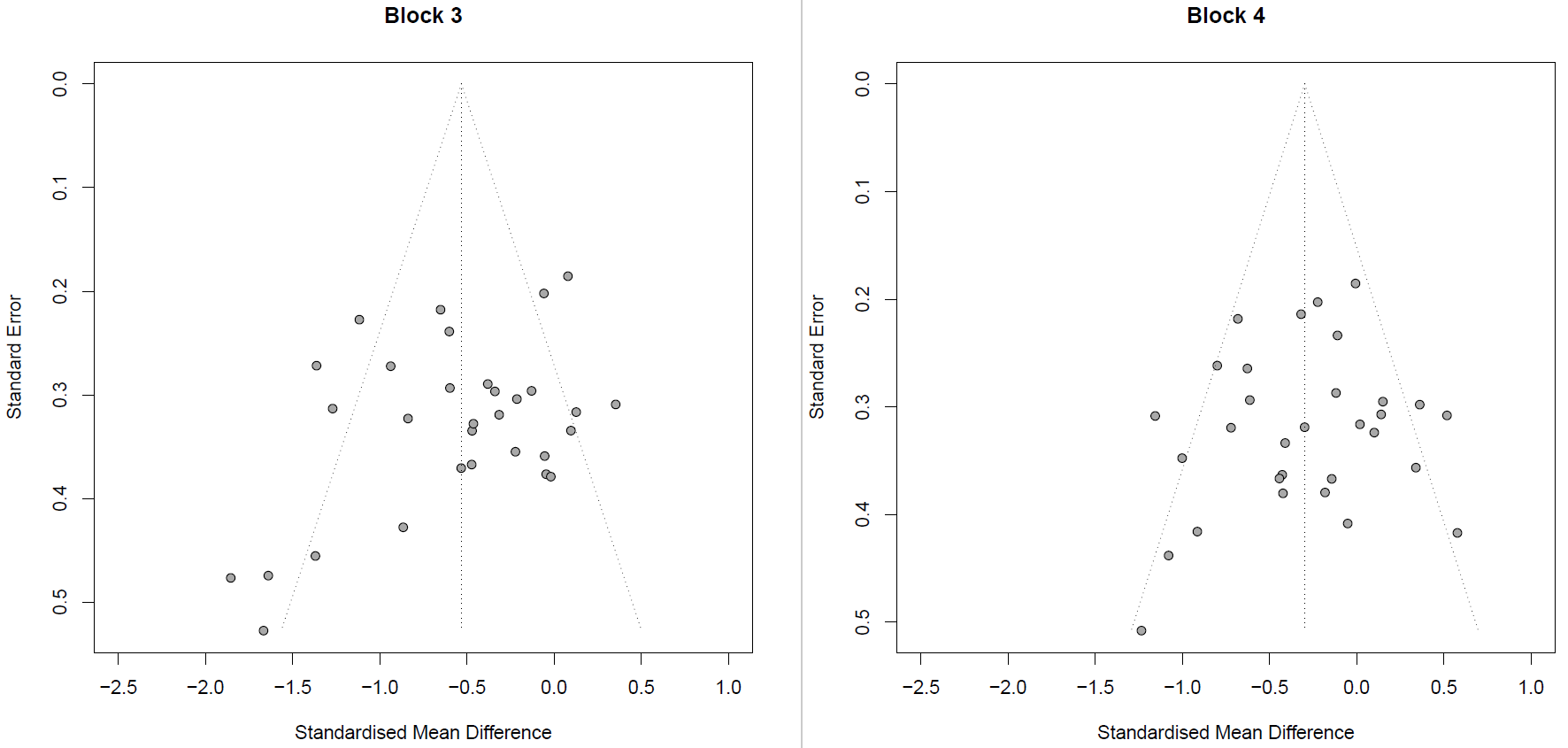


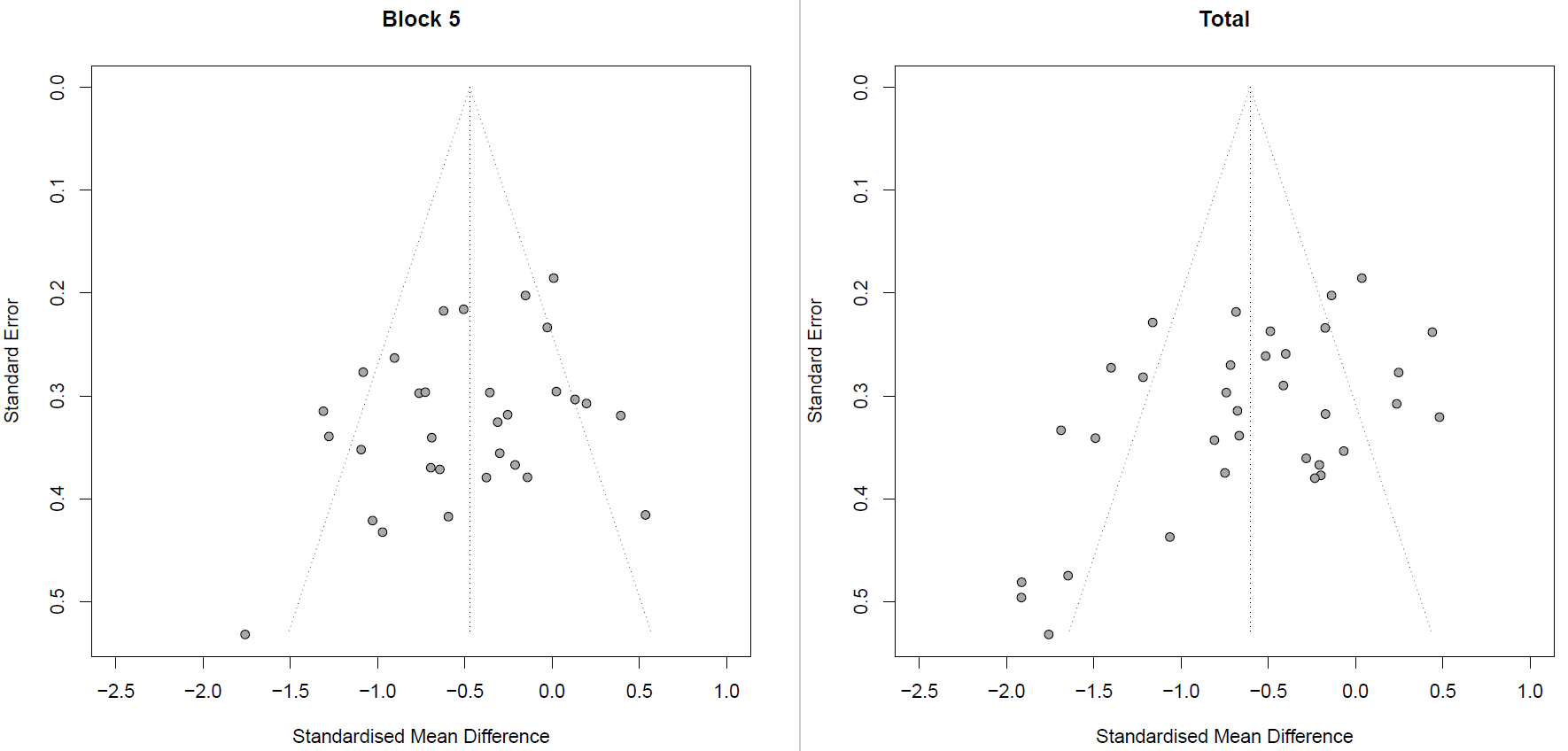

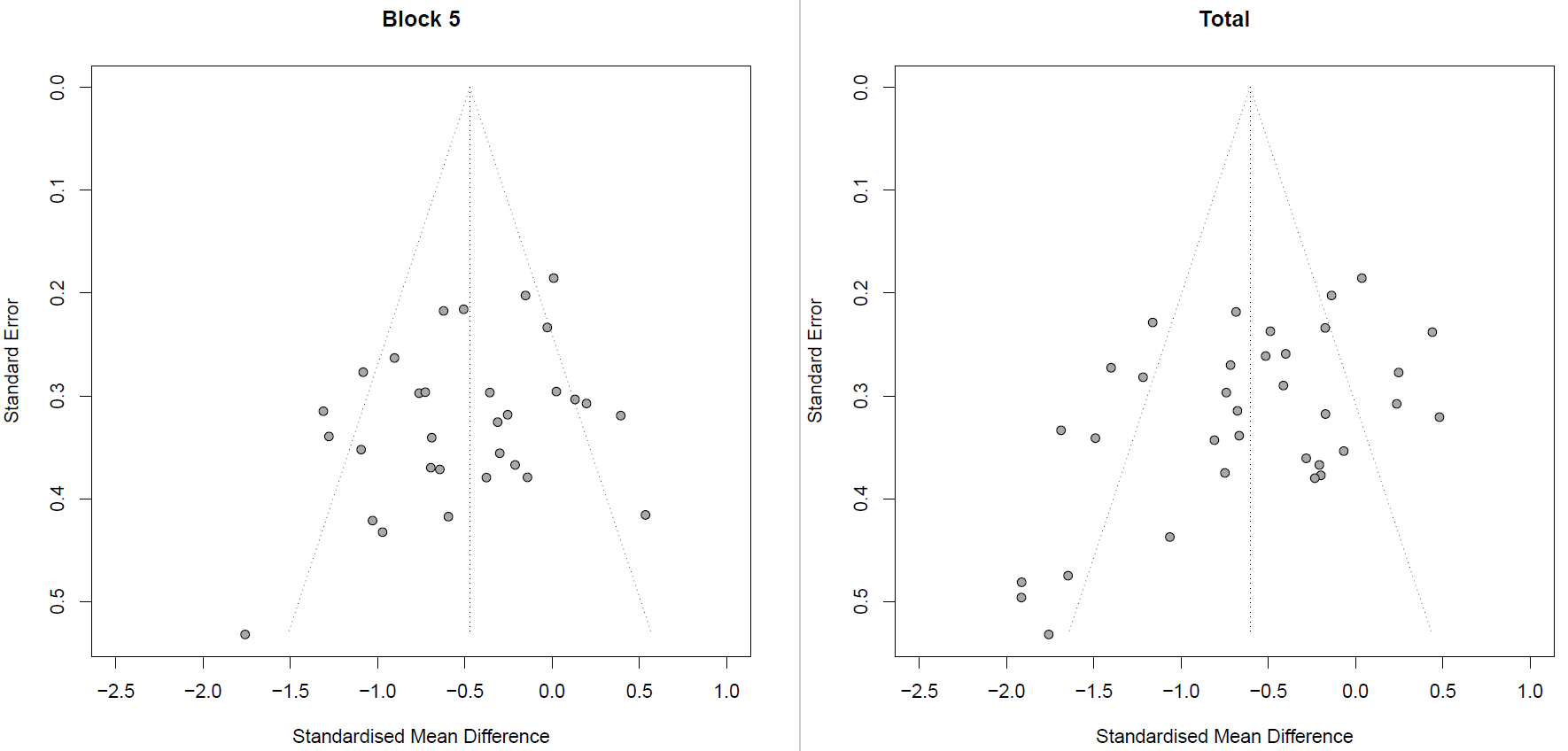


Note. Funnel plots based on standardised mean difference (g) in Iowa Gambling Task scores between individuals with brain injury and healthy controls. The dashed lines indicate the funnel. Each circle represents an individual study.

Table S6

*PRISMA 2020 for Abstracts Checklist*

| **Section and Topic** | **Item #** | **Checklist item** | **Reported (Yes/No)** |
| --- | --- | --- | --- |
| **TITLE** | | |  |
| Title | 1 | Identify the report as a systematic review. | YES |
| **BACKGROUND** | | |  |
| Objectives | 2 | Provide an explicit statement of the main objective(s) or question(s) the review addresses. | YES |
| **METHODS** | | |  |
| Eligibility criteria | 3 | Specify the inclusion and exclusion criteria for the review. | NO (due to word count limitations) |
| Information sources | 4 | Specify the information sources (e.g. databases, registers) used to identify studies and the date when each was last searched. | NO (due to word count limitations) |
| Risk of bias | 5 | Specify the methods used to assess risk of bias in the included studies. | NO (due to word count limitations) |
| Synthesis of results | 6 | Specify the methods used to present and synthesise results. | YES |
| **RESULTS** | | |  |
| Included studies | 7 | Give the total number of included studies and participants and summarise relevant characteristics of studies. | YES |
| Synthesis of results | 8 | Present results for main outcomes, preferably indicating the number of included studies and participants for each. If meta-analysis was done, report the summary estimate and confidence/credible interval. If comparing groups, indicate the direction of the effect (i.e. which group is favoured). | YES |
| **DISCUSSION** | | |  |
| Limitations of evidence | 9 | Provide a brief summary of the limitations of the evidence included in the review (e.g. study risk of bias, inconsistency and imprecision). | YES |
| Interpretation | 10 | Provide a general interpretation of the results and important implications. | YES |
| **OTHER** | | |  |
| Funding | 11 | Specify the primary source of funding for the review. | N/A |
| Registration | 12 | Provide the register name and registration number. | YES |

From: Page MJ, McKenzie JE, Bossuyt PM, Boutron I, Hoffmann TC, Mulrow CD, et al. The PRISMA 2020 statement: an updated guideline for reporting systematic reviews. BMJ 2021;372:n71. doi: 10.1136/bmj.n71

Table S7

*PRISMA 2020 Checklist*

| **Section and Topic** | **Item #** | **Checklist item** | **Location where item is reported** |
| --- | --- | --- | --- |
| **TITLE** | | |  |
| Title | 1 | Identify the report as a systematic review. | Page 1 |
| **ABSTRACT** | | |  |
| Abstract | 2 | See the PRISMA 2020 for Abstracts checklist. | Previous page of this document |
| **INTRODUCTION** | | |  |
| Rationale | 3 | Describe the rationale for the review in the context of existing knowledge. | Page 7-9 |
| Objectives | 4 | Provide an explicit statement of the objective(s) or question(s) the review addresses. | Page 9 |
| **METHODS** | | |  |
| Eligibility criteria | 5 | Specify the inclusion and exclusion criteria for the review and how studies were grouped for the syntheses. | Page 10-11 |
| Information sources | 6 | Specify all databases, registers, websites, organisations, reference lists and other sources searched or consulted to identify studies. Specify the date when each source was last searched or consulted. | Page 10 |
| Search strategy | 7 | Present the full search strategies for all databases, registers and websites, including any filters and limits used. | Page 10 |
| Selection process | 8 | Specify the methods used to decide whether a study met the inclusion criteria of the review, including how many reviewers screened each record and each report retrieved, whether they worked independently, and if applicable, details of automation tools used in the process. | Page 11-12 |
| Data collection process | 9 | Specify the methods used to collect data from reports, including how many reviewers collected data from each report, whether they worked independently, any processes for obtaining or confirming data from study investigators, and if applicable, details of automation tools used in the process. | Page 11-12 |
| Data items | 10a | List and define all outcomes for which data were sought. Specify whether all results that were compatible with each outcome domain in each study were sought (e.g. for all measures, time points, analyses), and if not, the methods used to decide which results to collect. | Page 12-13 |
|  | 10b | List and define all other variables for which data were sought (e.g. participant and intervention characteristics, funding sources). Describe any assumptions made about any missing or unclear information. | Page 12-13 |
| Study risk of bias assessment | 11 | Specify the methods used to assess risk of bias in the included studies, including details of the tool(s) used, how many reviewers assessed each study and whether they worked independently, and if applicable, details of automation tools used in the process. | Page 11-12 |
| Effect measures | 12 | Specify for each outcome the effect measure(s) (e.g. risk ratio, mean difference) used in the synthesis or presentation of results. | Page 14 |
| Synthesis methods | 13a | Describe the processes used to decide which studies were eligible for each synthesis (e.g. tabulating the study intervention characteristics and comparing against the planned groups for each synthesis (item #5)). | Page 10-12 |
|  | 13b | Describe any methods required to prepare the data for presentation or synthesis, such as handling of missing summary statistics, or data conversions. | Page 11-13 |
|  | 13c | Describe any methods used to tabulate or visually display results of individual studies and syntheses. | Page 15-16 |
|  | 13d | Describe any methods used to synthesize results and provide a rationale for the choice(s). If meta-analysis was performed, describe the model(s), method(s) to identify the presence and extent of statistical heterogeneity, and software package(s) used. | Page 14 |
|  | 13e | Describe any methods used to explore possible causes of heterogeneity among study results (e.g. subgroup analysis, meta-regression). | Page 14-16 |
|  | 13f | Describe any sensitivity analyses conducted to assess robustness of the synthesized results. | Page 15 |
| Reporting bias assessment | 14 | Describe any methods used to assess risk of bias due to missing results in a synthesis (arising from reporting biases). | Page 11 |
| Certainty assessment | 15 | Describe any methods used to assess certainty (or confidence) in the body of evidence for an outcome. | Page 13-14 |
| **RESULTS** | | |  |
| Study selection | 16a | Describe the results of the search and selection process, from the number of records identified in the search to the number of studies included in the review, ideally using a flow diagram. | Figure 1, Page 16 |
|  | 16b | Cite studies that might appear to meet the inclusion criteria, but which were excluded, and explain why they were excluded. | Page 16-17 |
| Study characteristics | 17 | Cite each included study and present its characteristics. | Tables 1, 2 and 3 |
| Risk of bias in studies | 18 | Present assessments of risk of bias for each included study. | Table 1 |
| Results of individual studies | 19 | For all outcomes, present, for each study: (a) summary statistics for each group (where appropriate) and (b) an effect estimate and its precision (e.g. confidence/credible interval), ideally using structured tables or plots. | Page 16-20, Table 4, Figure 2, Supplementary Materials S1 |
| Results of syntheses | 20a | For each synthesis, briefly summarise the characteristics and risk of bias among contributing studies. | Page 16-20, Table 1 |
|  | 20b | Present results of all statistical syntheses conducted. If meta-analysis was done, present for each the summary estimate and its precision (e.g. confidence/credible interval) and measures of statistical heterogeneity. If comparing groups, describe the direction of the effect. | Page 16-20 |
|  | 20c | Present results of all investigations of possible causes of heterogeneity among study results. | Page 16-20 |
|  | 20d | Present results of all sensitivity analyses conducted to assess the robustness of the synthesized results. | Page 16-20 |
| Reporting biases | 21 | Present assessments of risk of bias due to missing results (arising from reporting biases) for each synthesis assessed. | Page 19-20 |
| Certainty of evidence | 22 | Present assessments of certainty (or confidence) in the body of evidence for each outcome assessed. | Page 16-20 |
| **DISCUSSION** | | |  |
| Discussion | 23a | Provide a general interpretation of the results in the context of other evidence. | Page 20-23 |
|  | 23b | Discuss any limitations of the evidence included in the review. | Page 23-24 |
|  | 23c | Discuss any limitations of the review processes used. | Page 23-26 |
|  | 23d | Discuss implications of the results for practice, policy, and future research. | Page 23-26 |
| **OTHER INFORMATION** | | |  |
| Registration and protocol | 24a | Provide registration information for the review, including register name and registration number, or state that the review was not registered. | Page 9-10 |
|  | 24b | Indicate where the review protocol can be accessed, or state that a protocol was not prepared. | Page 9-10 |
|  | 24c | Describe and explain any amendments to information provided at registration or in the protocol. | 10 |
| Support | 25 | Describe sources of financial or non-financial support for the review, and the role of the funders or sponsors in the review. | Provided in a separate document in conjunction with paper guidelines |
| Competing interests | 26 | Declare any competing interests of review authors. | Provided in a separate document in conjunction with paper guidelines |
| Availability of data, code and other materials | 27 | Report which of the following are publicly available and where they can be found: template data collection forms; data extracted from included studies; data used for all analyses; analytic code; any other materials used in the review. | Provided in a separate document in conjunction with paper guidelines |

Note. From Page MJ, McKenzie JE, Bossuyt PM, Boutron I, Hoffmann TC, Mulrow CD, et al. The PRISMA 2020 statement: an updated guideline for reporting systematic reviews. BMJ 2021;372:n71. doi: 10.1136/bmj.n71

Figure S3

*Formulas used in the meta-analyses*

$$s_{p}= \surd\frac{\left( n_{1}-1 \right)s_{1}^{2}+\left( n_{2}-1 \right)s_{2}^{2}}{n_{1}+ n_{2}-2}$$

Pooled standard deviation

$$d= \frac{(\bar{X}_{1}-\bar{X}_{2})}{s_{p}}$$

Cohen's d

$$g=\left( 1-\frac{3}{{4(n}_{1}+ n_{2})-9} \right)d$$

Hedges’ g

$$V_{g}=(\frac{n_{1}+n_{2}}{n_{1}n_{2}})+(\frac{g^{2}}{2\left( n_{1}+ n_{2} \right)})$$

Sampling Variance of Hedges’ g

Table S8

Meta Regression of Total Scores – Without Imputation

| Model | *k* | *b* | *SE* | *p* | *95% CI* | Homogeneity statistics | |  |
| --- | --- | --- | --- | --- | --- | --- | --- | --- |
|  |  |  |  |  |  | *QE (df)* | *p* |  |
|  |  |  |  |  |  |  |  |  |
| % Male | 20 | -0.18 | 0.43 | 0.673 | [-1.02, 0.66] | 44.72 (16) | < .001*** |  |
| Average Age | 20 | -0.01 | 0.01 | 0.390 | [-0.03, 0.01] | 44.72 (16) | < .001*** |  |
| Average Education | 20 | 0.03 | 0.06 | 0.622 | [-0.09, 0.15] | 44.72 (16) | < .001*** |  |

*Note.* *k* = number of studies used in analysis, *b* = regression coefficient, *SE* = standard error, *p* = p value, *CI* = confidence interval, *QE* = QE statistic, *df* = degrees of freedom. For the results of this analysis with imputed values please see table S4. Please note sensitivity analysis revealed no differences between the imputed and non-imputed models.

****p* < .001
